# Developing an SMS text message intervention on sexual and reproductive health with adolescents and youth in Peru

**DOI:** 10.1186/s12978-020-00943-6

**Published:** 2020-07-31

**Authors:** Fiorella Guerrero, Nora Lucar, Mijail Garvich Claux, Marina Chiappe, Jose Perez-Lu, Michelle J. Hindin, Lianne Gonsalves, Angela M. Bayer

**Affiliations:** 1 Facultad de Salud Pública y Administración, Universidad Peruana Cayetano Heredia, Lima, Peru; 2 Reproductive Health Program, Population Council, New York, USA; 3 Department of Sexual and Reproductive Health and Research including the UNDP/UNFPA/UNICEF/WHO/World Bank Special Programme of Research, Development and Research Training in Human Reproduction (HRP), World Health Organization, Geneva, Switzerland; 4 Swiss Tropical and Public Health Institute (Swiss TPH), Basel, Switzerland; 5 University of Basel, Basel, Switzerland

**Keywords:** Adolescents, Youth, Sexual and reproductive health, Participatory approach, Focus groups, Text messaging, Peru

## Abstract

**Background:**

Improved access to sexual and reproductive health (SRH) services and information is essential for supporting adolescents and youth in making informed decisions and optimizing each young person’s outcomes related to their SRH, health and well-being and countries’ current and future social and economic development. Mobile phones offer opportunities for young people to privately access SRH content and to be linked to SRH services. The objective of this study was to develop the content for an SMS (short message service or “text message”) platform jointly with adolescents and youth in three regions in Peru (Lima, Ayacucho and Loreto) as part of the ARMADILLO (Adolescent/Youth Reproductive Mobile Access and Delivery Initiative for Love and Life Outcomes) Study.

**Methods:**

Content development was done in three stages. During Stage 1, we held community consultations with 13–17 year old adolescents, 18–24 year old youth and professionals who work with young people through the education and health sectors (“adult advisers”) to identify and rate SRH topics of interest through group free- and guided-brainstorming activities and an individual written sharing activity. During Stage 2, the team developed the preliminary domains, sub-domains and content for the SMS platform. During Stage 3, we held focus groups with adolescents to validate the SMS content, including both individual scoring of and group feedback for each SMS. Group feedback asked about their general impressions and understanding and their thoughts about the language and usefulness of the SMS.

**Results:**

A total of 172 adolescents and youth ages 13–24 and 20 adult advisers participated. Adolescents and youth brainstormed and rated SRH topics and sub-topics that led to the initial structure for the SMS platform, with 9 domains, 25 sub-domains and 146 draft SMS. Adolescents provided high scores for the SMS, with all sub-domains receiving average scores of 3.0 or higher (out of 4.0) for the SMS included. Adolescents also provided suggestions to optimize content, including improvements to unclear messages, resulting in SMS with adolescent-friendly content in simple, straightforward language. This process also revealed that adolescents lacked knowledge and had misconceptions related to contraceptive methods.

**Conclusion:**

This study details the systematic process used to develop relevant and accessible SRH information through a participatory approach. We document critical information about what young people know and how they think, enabling us to understand their perspective and literally speak their language. Results also provide future directions for programmatic, research and policy efforts with young people, in particular around gender norms, interpersonal violence, and access to SRH information and services, in similar settings.

## Plain English summary

Improvements to young people’s sexual and reproductive health (SRH) outcomes require the dissemination of information about SRH topics to young people to give them the tools and support to make informed decisions. Dissemination of information through text messages or SMS is a promising strategy given the privacy of SMS and the wide availability of cell phones among adolescents and youth.

In this study, we describe how we worked with Peruvian adolescents and youth to develop the structure and content for a pilot intervention to send SMS about SRH to young people in Peru. This study took place in three regions of Peru (Lima, Ayacucho and Loreto). We started with community consultations, where adolescents, youth and adult advisers brainstormed about SRH topics of interest to young people. In a second stage, the team developed an initial SMS platform structure and several draft SMS. In the third stage, we held focus groups to ask adolescents for their opinions about the SMS. In this way, young people participated actively in the entire process.

Our findings show what young people know and how they think about SRH topics, enabling us to understand their perspective and literally speak their language to provide them with information that they need and that they will receive, find approachable and useful, and ultimately, take into consideration in order to make good decisions. Results also provide future directions for programmatic, research and policy efforts with young people, in particular around gender norms, interpersonal violence, and access to SRH information and services in similar settings.

## Introduction

Globally, there are approximately 1.9 billion adolescents and youth ages 10–24 who represent about one-fourth of the total population [1]. In Peru, there are approximately 8.7 million adolescents and youth ages 10–24 who represent 28% of the total population [2]. Considering the significant size of this population, it is critical to invest in them to improve their knowledge and skills, including those related to sexual and reproductive health (SRH), in order to optimize both each young person’s development, SRH outcomes and other outcomes related to their health and well-being, and Peru’s current and future social and economic development.

Although the prevalence of adolescent pregnancy has declined worldwide, in Peru, adolescent pregnancy remains unchanged over time and continues to be unintended for the majority of adolescents. According to the 2017 Peru Demographic and Health Survey (DHS/ENDES), 13.4% of 15–19 year old females were currently pregnant or parenting [3], a percentage that has not changed significantly since 1991/92 [4]. Pregnancy is higher among adolescents living in rural versus urban areas (23% vs. 11%), and those living in the jungle (23%) versus the highlands or coast (13 and 12%). Also, pregnancy is higher among those who have primary versus secondary education (45% vs. 12%), and those in the poorest versus wealthiest economic quintile (24% vs. 4%). In 2016, 66% of Peruvian 15–19 year olds who had been pregnant reported that their last pregnancy was unintended (58% mistimed and 8% unwanted). Reported unintended pregnancies have steadily increased over time, up from 47% in 1991/2 to 55% in 2000 [5]. This is in contrast to the group of 20–24 year old females, among whom the age-specific fertility rate has declined from 200 births per 1000 females in 1991/92 to 112 per 1000 in 2015/16 [6].

Sexually transmitted infections (STIs) including the human immunodeficiency virus (HIV) is another SRH issue that impacts Peruvian youth. A large population-based study with 18–29 year old youth in smaller Peruvian cities, not including the capital Lima, resulted in the following prevalence estimates: 6.5% in women and 4.2% in men for chlamydia; 4.9% in women and 0.3% in men for trichomonas; 0.4% in women and 0.5% in men for syphilis; 0.1% in both women and men for gonorrhea; and 0.1% in women and 0.5% in men for HIV [7].

Although Peru offers “adolescent-friendly” health services – including SRH services – they are not as friendly for adolescents as they are proposed to be. The Peruvian Ministry of Health (MOH or MINSA) has worked to create and expand adolescent-friendly health services for adolescents, from 213 health establishments in 2006 to over 2800 establishments in 2016. MINSA states that these services offer adolescent-friendly hours, providers and services, including services for SRH [8]. However, a recent oversight report by the Peruvian Ombudsman’s Office (*Defensoría del Pueblo*) found that these adolescent-friendly health services were not actually tailored to adolescents’ needs [9]. Additionally, services also required parental accompaniment, running counter to current legislation [9]. One-quarter (25%) of health establishments require the accompaniment of parents or guardians for minors to access services, despite the fact that the Family Planning Health Technical Guideline no longer requires it as of August 2017 [10].

Given the above challenges faced by adolescents in accessing needed SRH services and information, it is critical to develop innovative strategies to improve adolescents’ access to relevant and reliable information through channels outside of health services. One such channel is mobile phones, through which adolescents can easily access information about different SRH topics, including other services available to them through the public health and other systems. The high penetration of mobile phones globally, including among adolescents and youth, further affirms the potential of this alternative channel. For instance, among 15–29 year old Peruvians, mobile phone ownership is higher than in the general population, with 71% of Peruvian young people reporting ownership [11].

There have been several interventions to provide SRH information to young people in different upper-income countries and low- and middle-income countries (LMICs) using mobile phones. For instance, the mCenas! program used an interactive SMS (short message service or “text message”) system to provide narrative and informational messages to 15–24 year olds in Mozambique to increase their knowledge about contraceptive methods and address common related myths and barriers [12]. The Learning about Living Program used a website to provide prevention education regarding maternal health, HIV/AIDS and gender-based violence to 10–21 year olds in Nigeria [13]. The Access, Service & Knowledge (ASK) initiative used mobile and electronic platforms to disseminate information on SRH and HIV/AIDS to 10–24 year olds in five African and two Asian countries [14]. YoungAfricaLive used a community mobile platform to provide information about love, sex and relationships to young people in Kenya, South Africa and Tanzania, also taking into account their social, cultural and economic situation [15]. These studies demonstrated increased contraceptive knowledge [16] and improved knowledge and attitudes toward sexuality indicators such as HIV/AIDS and gender-based violence [13] among youth participants. However, there is limited information available about formative phases and many programs have failed to incorporate young people’s perspectives into the development of program content, despite international guidelines about how the integration of young people’s voices can increase program effectiveness [17]. Similarly, few of these interventions have been rigorously evaluated and evidence on the feasibility and effectiveness of adolescent-and-youth-targeted mobile phone-based SRH interventions is limited.

To address these research gaps, the World Health Organization (WHO), the Universidad Peruana Cayetano Heredia (UPCH) and the International Centre for Reproductive Health Kenya (ICRH-K) initiated “ARMADILLO” (Adolescent Reproductive Mobile Access and Delivery Initiative for Love and Life Outcomes) with the overall objective of generating evidence on the effect of access to SMS-based SRH information on health-related outcomes among adolescents. Adolescent-targeted mHealth interventions should be both developed and tested with young people in order to identify and incorporate appropriate SRH information content and system design [18, 19] and to document successful strategies to meaningfully engage young people. The specific objective of the first phase of ARMADILLO was to develop the ARMADILLO SMS (text message) platform, including the SRH topics and sub-topics, the SMS system design and the SMS content, using a participatory approach with young people. This manuscript documents the participatory process used in Peru, which consisted of a three-stage effort to develop the platform in collaboration with adolescents and youth ages 13–24. The process and the findings may be useful to inform other initiatives, especially in Latin American countries, that aim to address the SRH challenges faced by adolescents and youth.

## Methods

The development of the ARMADILLO content included three stages to develop the SMS platform together with adolescents and youth: (1) work with adolescents and youth to identify and rate the topics that are of greatest interest to them and other adolescents and youth; (2) develop the initial structure of the SMS platform, including topics, sub-topics, and the SMS; and (3) validate those initial SMS with adolescents. (See [20] for more detail on ARMADILLO.)

### Study context

The formative research for ARMADILLO was carried out in three locations in Peru, a country with an estimated population of 32 million [21]. The study was implemented in urban and peri-urban districts of the country’s three natural regions: Lima, the capital city located on the coast; Ayacucho, in the mountains; and Loreto, in the jungle. These regions were also selected since they have high rates of adolescent pregnancy, as follows: Metropolitan Lima, 8.3%; Ayacucho, 13.8%; and Loreto, 25.6% [22].

### Data collection

The three stages of the study were carried out between November 2015 and July 2016.

#### Stage 1. Community consultations with adolescent and youth participants and professionals for identification of topics of interest to young people

The first stage in message development consisted of informal, community consultations. These included adolescent and youth participants and professional “adult advisers” in the three study sites. The study team contacted several adults in each community (“gatekeepers”) who had worked extensively with adolescents and youth. These gatekeepers invited young people from schools, health establishments and the general community to participate, to obtain a diverse sample of participants that included in- and out-of-school adolescents, youth who were working and not working, and some expectant and parenting young people. Adult advisers were adults who engage closely with young people in their professional work, including education professionals such as school officials and teachers, health professionals such as midwives and psychologists, and community organizers. Gatekeepers also recruited adult advisers who served primarily as observers.

We conducted six community consultations in the three study sites, with two meetings at each site with the following structure: one meeting with 13–17 year old females and males and an adult adviser; and one meeting with 18–24 year old females and males and an adult adviser. The ideas and perspectives of young people were prioritized, and adults contributed their ideas only after adolescents and youth had finished. Each community consultation was facilitated by AMB and a second member of the team. Meetings were conducted in Spanish and lasted 1.5–2 h.

The community consultations were designed to generate and rank SRH topical areas. First, participants were given the following prompt for a free-brain-storming: “Thinking about sexual and reproductive health, what are the topics or things that are of most interest to you and other adolescents and youth you know?” After no new ideas emerged, the facilitator initiated a guided-brainstorming by mentioning several SRH topics that had not been mentioned by participants in the free-brainstorming and that had been included in other similar SRH projects and studies with young people in other contexts [23–27] and in Peru [28–30]. For each topic mentioned, the participants worked to reach a consensus about whether the topic was “not of interest,” “of interest,” or “of great interest” to them. Participants selected a notetaker who wrote all responses on butcher paper and were recorded without attribution to a specific participant. Finally, participants were given a sheet of paper to write down any other SRH topics that may be of interest to them to facilitate the private expression of ideas that might have been omitted during the group activities because of shame, fear of being judged or other reasons. These topics were then added to the free-brainstorming list on the butcher paper. In addition to the notes on the butcher paper and the individual notes from participants, the team also took detailed notes about all of the conversations during the community consultations. None of the notes recorded any identifying information about participants.

#### Stage 2. Development of the initial SMS platform structure and draft SMS content by the team

The initial structure of the platform was developed in two steps. First, four members of the team separately reviewed the brainstormed topics and scores from stage 1 to create a possible structure for the platform, including domains (topics) and sub-domains (sub-topics). Second, the team members came together to share their ideas and create a joint proposal of domains and sub-domains. Afterwards, the team developed the SMS, creating 2–10 SMS for each sub-domain of the platform. The team carefully reviewed all of the documentation from the community consultations in stage 1, including the topics of interest and the language that the adolescents and youth participants used when talking about these topics, to draft the SMS content. SMS could contain up to 140 characters, due to the characteristics of Peruvian mobile phones. A total of 146 SMS were developed.

#### Stage 3. Focus groups with adolescents for review and validation of proposed SMS

To validate the SMS content, we used purposive sampling (with close assistance from adult gatekeepers described in stage 1) to recruit adolescents from the three study sites to participate in 12 focus groups. We conducted four focus groups at each site with the following structure: two groups with 13–15 year old females and males; and two groups with 16–17 year old females and males. We included only 13–17-year olds in this stage in response to national stakeholders’ desire to see a special focus on this period of adolescence, given Peru’s adolescent pregnancy and early sexual debut statistics. Participants in stage 3 had not participated in stage 1.

For the focus groups, each group of participants evaluated 36–37 SMS (146 SMS in total) and all SMS were reviewed by each study site. At each focus group, every SMS was reviewed one by one, with an individual evaluation followed by a group evaluation of the SMS. For the individual assessment, each adolescent received a form to rate each SMS. The individual assessment consisted of a single question, “What do you think of this text message?”, with Likert scale response options accompanied by sad and smiling faces in four points ranging from very bad (1 point) to very good (4 points). For the group assessment, each SMS was projected on the wall and group opinions were requested. The facilitator generated dialogue by asking about: participants’ general impression of the message (“What do you think of this message? If this message came to you, what would you think?”); their understanding of the message (“If you had to explain what the message says, what would you say?”); the language of the message (“Do you understand the language of the message? Are we using appropriate words?”); and the usefulness of the message for the participants (“How can this message be improved? Is there anything you don’t like about the message or that you think young people you know wouldn’t like?).”

The focus groups were facilitated by a young researcher with experience carrying out social science and qualitative research with young people in Peru. The meetings were conducted in Spanish and lasted 1.5–2 h. A second member of the team took detailed notes of all comments for each of the SMS for further analysis and incorporation of youth feedback into each SMS.

### Data management and analysis

All data management and analyses were conducted in Microsoft Excel (Microsoft Corp., Redmond, WA, USA). For stage 1, we created a master list of all of the topics mentioned during the community consultations and assigned a score between 0 and 3 to each topic, according to the participants’ opinions: 3 for topics that participants mentioned spontaneously during the free-brainstorming or individual sharing; 2 for topics that participants rated as “of great interest” during the guided-brainstorming; 1 for topics that participants rated as “of interest” during the guided-brainstorming; and 0 for topics that participants did not mention or that participants said were of “no interest” during the guided-brainstorming. Sub-groups of participants were established based on age coupled with study site (e.g., adolescents aged 13–17 from Lima; youth aged 18–24 from Yurimaguas). A column was created for each sub-group of participants. Scores were generated for each sub-domain and for each domain, which is the mean of all sub-domains within that domain, using the sub-domains and domains developed during stage 2. These scores were developed for each sub-group of participants, for 13–17 and 18–24 year-olds overall.

For stage 3, we calculated the average score for each SMS, for each study site and overall. We also calculated the average score for each domain and sub-domain of the platform. All participant comments regarding each SMS were also consolidated in Excel. All of the comments for each SMS were analyzed in order to synthesize the main messages and integrate them into the final content and wording of the SMS. Two members of the team independently analyzed all participant comments, wrote up the primary take away messages, and drafted a revised SMS. Then, the entire team met to: discuss the take away messages; go back to review the comments again if there were discrepancies between the two reviewers; review the revised SMS proposals; and develop a final version of the SMS.

### Ethical considerations

All adolescent and youth participants provided their written informed consent or assent (depending on whether they were over 18) prior to participating in community consultations or research activities, such as focus groups. The parents and guardians of participants under age 18 also provided their written informed consent for their child’s participation. If participants disclosed instances of having experienced or witnessed violence or indicated mental distress during any activity, they were provided with information regarding services for general and mental health and violence in their communities, including services that are specifically for young people. The ARMADILLO study was approved by the World Health Organization Research Ethics Review Committee and the Universidad Peruana Cayetano Heredia’s ethics committee.

## Results

### Stage 1. Community consultations with adolescent and youth participants and professionals to identify topics of interest to young people

A total of 68 13–24 year olds participated in the six community consultations (see Table 1). In the three consultations with 13–17 year old adolescents, 69% were females and 31% were males. In the three consultations with 18–24 year old youth, 64% were females and 36% were males. In each site there were parenting or expecting adolescent and youth participants. Twenty adults also participated: Lima [3]; Yurimaguas [5]; and Ayacucho [12].

**Table 1:** Adolescent and youth participants in Stage 1 community consultations, by age group and sex, Lima, Yurimaguas and Ayacucho, Peru, 2015

	Adolescents Aged 13–17		Youth Aged 18–24	
	Females	Males	Females	Males
**Lima**	7	6	4	7
**Yurimaguas**	4	3	4	5
**Ayacucho**	11	1	15	1
**Total**	22	10	23	13

Adolescent and youth participants named many topics of interest, which were later grouped into broad SRH topics and narrower sub-topics. Adolescents demonstrated a lack of knowledge regarding different SRH terminology and, in particular, regarding contraceptive methods. For example, they were only aware of emergency contraception pills, also known as the “morning after” or “day after” pill, which are for use in emergency or unanticipated situations, and were unaware of regular hormonal contraceptive pills, which are for use as regular birth control. They were also unaware of long-acting reversible contraceptive methods (LARCs), such as intrauterine devices (IUDs) and implants, or of their benefits over other contraceptive methods. Once informed during the guided-brainstorming, participants expressed fear about possible negative side effects of regular contraceptive methods and particularly LARCs, including sterility, and considered them inappropriate for adolescents. Even the condom was not well understood, as it was perceived as the best method to prevent pregnancy but not as the best method to prevent STIs.

The ten SRH topics and respective sub-topics that resulted from the community consultations are shown in Table 2, presented according to the rating assigned to them by the adolescents and youth participants. The highest scored topics were identity, STIs, and specific barrier and contraceptive methods. The lowest scored categories were alcohol and drugs, SRH rights and policies, and pregnancy. The average scores ranged from 0.2 to 1.7 out of a possible 3.0. One of the main drivers for lower scores was lack of interest in certain sub-domains. For example, ‘violence’ as a topic had an average score of 1.2 because participants indicated little interest in the sexual abuse and rape sub-domains (scores of 0.4), but higher interest in the intimate partner violence sub-domain (score of 2.0).

**Table 2:** Adolescent and youth participants’ level of interest in different sexual and reproductive health (SRH) topics and sub-topics (Stage 1), by age group, Lima, Yurimaguas and Ayacucho, Peru, 2015

	Interest in sub-topics according to score, range from 3 (great interest) to 0 (no interest)^**1,2**^			
**Topics (Total average score)**	**Adolescents aged 13–17**	**Average score**	**Youth aged 18–24**	**Average score**
**Identity (1.7)**	Self-esteem	(2.5)	Exploration of sexuality	(3.0)
	Sexual identity	(2.3)	Menstruation	(2.7)
	↓ Physical changes in males	(1.0)	Sexual orientation	(2.3)
	↓ Physical changes in females	(1.0)	↓ Physical changes in males	(1.0)
			↓ Physical changes in females	(1.0)
**Sexually transmitted infections (STIs) (1.7)**	HIV	(2.3)	STI symptoms	(2.0)
	↑ STI prevention	(1.5)	↑ STI prevention	(2.0)
	↓ STI symptoms	(1.0)	↑ Herpes	(2.0)
	O Herpes	(0.0)	↓ HIV	(1.0)
**SRH barrier and contraceptive methods (for preventing pregnancy, STIs and HIV) (1.6)**	Abstinence	(2.3)	Rhythm	(3.0)
	↑ Rhythm	(1.5)	Withdrawal	(3.0)
	↓ Withdrawal	(0.5)	↓ Abstinence	(1.0)
	Emergency contraception (“morning after pill” or “day after pill”)	(3.0)	Emergency contraception	(3.0)
	Male condom	(3.0)	Male condom	(3.0)
	↓ Intrauterine devices (IUDs)	(0.8)	↑ IUDs	(2.0)
	↓ Implants	(0.8)	O Implants	(0.0)
**Interpersonal relationships (1.3)**	Family comunication (in general and related to sexuality)	(3.0)	“Friends with benefits”	(2.7)
	Peer pressure: sexual intercourse	(2.5)	Peer pressure: sexual intercourse	(2.7)
	↓ Sexual exploration with partner	(0.8)	O Attraction and flirting	(0.0)
	O Attraction and flirting	(0.0)		
**Sexual practices (1.3)**	Oral sex	(2.5)	Transactional sex	(2.7)
	Transactional sex	(2.3)	Anal sex	(2.3)
	↑ Masturbation	(2.0)	Oral sex	(2.3)
	O Sex without penetration	(0.0)	↑ Masturbation	(2.0)
	O Mutual masturbation	(0.0)	↓ Sex without penetration	(0.3)
			↓ Mutual masturbation	(0.3)
**Violence (1.2)**	Intimate partner violence	(2.5)	↑ Intimate partner violence	(2.0)
	Sexual abuse and rape	(2.3)	↓ Sexual abuse and rape	(0.4)
	O How to prevent violence	(0.0)	↓ How to prevent violence	(0.3)
**Health services (1.1)**	↑ SRH services	(1.3)	Access to health services	(3.0)
	↓ Access to health services	(0.3)	↑ Mental health /psychological services	(2.0)
	O Health insurance	(0.0)	O Health insurance	(0.0)
**Pregnancy (0.7)**	Pregnancy	(3.0)	Pregnancy	(3.0)
	O Pregnancy signs	(0.0)	↓ Pregnancy signs	(1.0)
	O Pregnancy complications	(0.0)	O Pregnancy complications	(0.0)
**SRH rights and policies (0.6)**	Abortion	(2.3)	Abortion	(3.0)
	O Right to information on SRH	(0.0)	↓ Right to information on SRH	(0.3)
	O Right to health care	(0.0)	↓ Right to health care	(0.3)
**Alcohol and drugs (0.2)**	↑ Types of drugs	(1.3) (0.8)	↓ Peer pressure: Alcohol and drug usage	(0.4)
	↓ Peer pressure: Alcohol and drug usage		O Types of drugs	(0.0)

1 The scores in the Table are based on adolescents’ and youth’s ratings of the SMS text messages during the Stage 1 community consultations. Participants both a) spontaneously mentioned topics and sub-topics of interest and b) rated their interest in sub-topics included in SRH projects in other settings. The rating scale ranges from 0 (of no interest) to 3 (of great interest). For the sub-topics, the score represents the average rating of the sub-topic across the six meetings. For the topics, the score represents the average of the scores for the sub-topics included under the topic. Only the highest and lowest scored sub-topics are included in the Table. / 2 The symbols to the left of the sub-topics mean the following: O - Of no interest (Equal to zero); ↓ - Of little interest (Score between 0.1 and 1.0); ↑ - Of interest (Score between 1.1 and 2.0); and  - Of great interest (Score between 2.1 and 3.0)

Interest in SRH topic differed by age (see Table 2). Adolescent participants showed greater interest in self-esteem, abstinence, and HIV and gave the highest rating to communication with their families, both in general and about sexuality-related topics. Older youth participants showed greater interest in topics such as exploration of their sexuality, STI symptoms and prevention, “friends with benefits” (friends that may engage in sexual activity without considering themselves “in a relationship”), peer pressure around sex, and access to health services including psychological and mental health services. Both age groups were interested or very interested in contraceptive methods (in general and each method specifically), intimate partner violence, pregnancy, and abortion.

When participants were given the opportunity to write down topics of interest, detailed questions about doubts and curiosities related to SRH, identity and sexual orientation emerged. For example, “Why do some people like others from their same sex?”, “I would like to know more about hermaphrodites”, or “Is it good or normal to feel love for a man, if I’m a man?”. Questions also emerged about psychological, physical and sexual abuse in the family environment, including “How can I cope with violence and alcoholism of parents?” or “What can be said to a teenager who has been raped several times by her stepfather at age 6, who currently feels despised by her mother and wishes to die?”

### Stage 2. Development of the initial structure of the SMS platform and draft SMS by the team

**Fig. 1:**
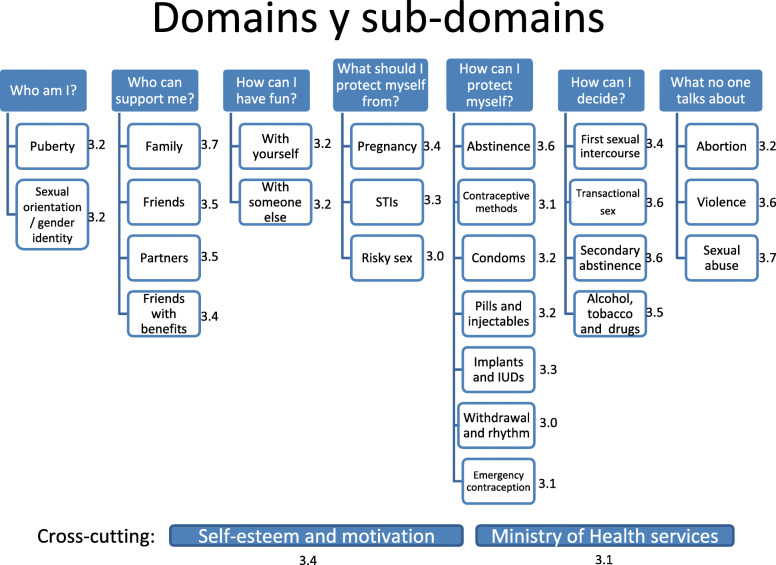
Design of the domains and sub-domains for the ARMADILLO Peru SMS platform and average score for adolescents’ ratings of the SMS messages included in each sub-domain, Lima, Yurimaguas and Ayacucho, Peru, 2016^1^

The team developed a potential structure for the platform based on an in-depth, critical analysis of the information shared by stage 1 participants. The final draft structure of the platform was classified into nine domains or themes. (See detail in Fig. 1.) Seven of these domains were “independent”, in that all of the SMS related to that topic were included in the domain. Independent domains were 1) who am I?; 2) who can support me?; 3) how can I have fun?; 4) what should I protect myself from?; 5) how can I protect myself?; 6) how can I decide?; and 7) what no one talks about. The team decided to phrase the domains as questions, instead of as statements, to try to appeal to young people and have the platform “speak” to the questions they might have. The other two domains were crosscutting, in that the messages referred to themes that could be relevant for many of the independent domains: 8) self-esteem and motivation; and 9) access to Ministry of Health services. Additionally, the SMS included in these cross-cutting domains would be distributed interspersed with each of the independent domains.

The initial structure also included 25 sub-domains or sub-themes, with 2–7 sub-domains for each domain, depending on the complexity of the domain itself. Each sub-domain had 2–10 messages. The language of the messages was intentionally drafted to be conversational, rather than authoritative, as well as simple (at the level of an adolescent) and colloquial, following the model of similar popular SMS-based services already in place.

### Stage 3. Focus groups to review and validate the SMS with adolescents

A total of 104 adolescents participated in the twelve focus groups in the final stage. This included six focus groups with 50 13–15 year olds and six focus groups with 54 16–17 year olds. Across all participants, 52% were females and 48% were males (Table 3).

**Table 3:** Participants in Stage 3 focus groups, by age group and sex, Lima, Yurimaguas and Ayacucho, Peru, 2016

	Ages 13–15		Ages 16–17	
	Females	Males	Females	Males
**Lima**	10	9	9	9
**Yurimaguas**	9	8	8	9
**Ayacucho**	9	5	9	10
**Total**	28	22	26	28

#### Results of adolescents’ individual assessment to rate their general impressions of the SMS

Figure 1 presents the average score for all of the SMS included in each sub-domain. In contrast to the ratings of the SRH topics during stage 1, many of which were low, the SMS received uniformly high ratings. The highest-rated sub-domains were: the family (3.7); sexual abuse (3.7); abstinence (3.6); transactional sex (3.6); secondary abstinence (3.6); violence (3.6); friends, partners, alcohol, tobacco and drugs (all with 3.5). The remaining sub-domains also received high ratings, of 3.0–3.4.

#### Results of adolescents’ group assessment to provide their perspectives regarding the ease of understanding, language and usefulness of the SMS

Regarding wording, participants had different types of suggestions for improving the acceptability and understanding of the messages. These suggestions were similar across age groups. One of these was the suggestion to adapt messages by sex. Adolescents stated that they felt that some messages were inappropriate for females or for males and that some messages should be addressed only to the group to which the message applied; for instance, that females should not receive information about male puberty and vice versa. Additionally, they indicated that some topics were new to them: for example, female adolescents from both age groups were previously unfamiliar with the topic of female masturbation. Therefore, a related suggestion was to make sure that the messages were very specific so that adolescents would have a good understanding of the content.

Adolescent participants also suggested further refining the tone of certain messages and using more familiar language to capture the attention of adolescents. For example, they suggested changing the use of the imperative (for example, “Do this to be able to …” ) to more neutral language (for example, “You can do this to be able to …” ). They also recommended the use of words preferred by youth, such as “boyfriend/girlfriend” instead of “intimate partner,” “likes” instead of “preferences,” or “v zone” instead of “vagina.” They also recommended adding phrases like “no kidding” and exclamation points to specific messages, to ensure that adolescents believe and take the content seriously.

Table 4 shows a selection of ARMADILLO text messages from different domains and sub-domains at two different points during this formative research effort: the draft version of the SMS, as created by the team during stage 2 based on the findings from stage 1; and the final version of the SMS, after integrating all of the feedback that the adolescents provided for each SMS during stage 3. As shown in the Table, some messages changed minimally while others changed drastically. For SMS with minimal changes, participants often wanted to add greater emphasis to certain text in the message, for example, by capitalizing the word “no” or “not” or by adding an exclamation point for extra emphasis. For SMS with more changes, participants wanted to modify the language to make it more adolescent-friendly or to make it more specific.

**Table 4:** Examples of draft and final versions of SMS text messages from different domains and sub-domains of the ARMADILLO Peru SMS platform

Domains and *Sub-domains*	Sample text message^**1**^ from sub-domain, draft version created by team during stage 2	Sample text message^**1**^ from sub-domain, final version following participant feedback during stage 3
**Who am I?** *Puberty*	“During adolescence the body changes drastically. It’s normal that for some people, these changes are more obvious than for others.”	“During adolescence the body has many physical and emotional changes called puberty. We do NOT all go through the same changes!”
**Who am I?** *Sexual identity*	“We are all born with one sex (man or woman) but not all of us feel good about it. It’s normal! A person is what he or she feels.”	“We are all born with one sex (man or woman) but not all of us feel good about it. It’s normal! The decision is yours.”
**Who can support me?** *Family*	“Nobody is taught to be a parent. Sometimes they want to help you and give you their love, but they do not always know how. Look for moments.”	“Nobody is taught to be a parent. Sometimes they want to help you and give you their love, but they do not always know how to do it. Ask them for support and they will support you!”
**Who can support me?** *Friends*	“A friend does not pressure you to do something you do not want to do. A friend respects your decision.”	“A friend does NOT pressure you to do something you do not want to do. A friend respects your decision.”
**Who can support me?** *Partners*	“A healthy relationship is one that makes you happy. A relationship that only makes you sad isn’t worth it.”	“A healthy relationship is one that makes you happy, where there is mutual appreciation, fidelity, respect and good communication.”
**How can I have fun?** *With someone else*	“To be able to enjoy sex with someone, trust and acceptance are key.”	“Before having sex with someone, you should feel comfortable with that person and also with yourself. Don’t forget to use protection!”
**What should I protect myself from?** *Pregnancy*	“A pregnancy can happen at any time. Even the first time.”	“A pregnancy can happen at any time. Even during your first sexual intercourse. It is true. Be aware!”
**What should I protect myself from?** STIs	“If you have had oral, vaginal or anal sex without a condom, get tested for STIs. If you know that your partner has or had an STI, with even more reason.”	“If you have had sex without a condom, get tested for sexually transmitted infections or STIs. Find out more about the topic. The test is FREE.”
**How can I protect myself?** *Abstinence*	“Deciding not to have sex until you find the right person and the right time can be a good decision.”	“Deciding not to have sex until you find the right person and feel prepared is a responsible decision. There’s no rush!”
**How can I protect myself?** *Condom*	“Condoms are not only for vaginal sex. They are also for anal and oral sex. And use a new condom for every erection.”	“The condom should be used for vaginal, anal and oral sex. Remember to use a new condom for every erection!”
**How can I protect myself?** *Implants and IUDs*	“The implant and intrauterine device or IUD are the most effective methods to prevent pregnancy. And they are appropriate for teenagers.”	“The implant and intrauterine device or IUD are the MOST EFFECTIVE methods to prevent pregnancy. And they are appropriate for teenagers!”
**What no one talks about** *Abortion*	“Having an abortion in an informal place or one that offers to resolve ‘menstrual delay’ can lead to the death of the woman.”	“Having an abortion in a clandestine place can cause health problems or even death.”
**What no one talks about** *Violence*	“The more I hit you, the more I love you? NO. No one who hits you can love you. No one who makes you suffer can love you.”	“The more I hit you, the more I love you? This is not right. No one who hits you can love you. No one who makes you suffer can love you. VALUE YOURSELF! “

^**1**^Each sub-domain had 2–10 text messages associated with it

## Discussion

This manuscript is one of the first to document participatory programmatic and research processes for building text messaging systems for public health interventions together with adolescents and youth. Limited past evidence has shown that SRH behavioral change interventions developed together with adolescent and youth participants are better received [31, 32]. For instance, in a study in the UK that aimed to reduce the incidence of STIs through an SMS-based intervention, the authors found that messages that were tested before initial deployment through focus group discussions with young people aged 16–24 later showed that all of the messages were considered easy to understand and that none of the messages were rated poorly [31].

We adopted a similar participatory approach for ARMADILLO intervention’s development process. The research was preceded by a consultative stage 1, where adolescent and youth participants decided the topics they wanted to be included in the SMS platform. During a preparatory stage 2, the team developed the initial system based fully on these community consultations. The messages adolescent participants reacted to in stage 3, therefore, were thoughtfully crafted based on an extensive initial consultation. During data collection focus groups, adolescent participants gave their individual and group opinions about each potential SMS for the ARMADILLO platform. The results of ARMADILLO’s participatory process provide insights into what Peruvian young people prefer, understand, and accept from proposed SRH digital intervention content. Separately, they also reveal important health-related information, norms, and behaviors where more attention (digital or otherwise) is needed.

Young people's feedback affirmed what previous work with adolescents found: that language should be simple; allow adolescents to relate the contents with personal experiences; and have a reliable, friendly, professional tone [31]. We also showed that importance and relevance are perceived differently for broad topical areas (i.e. topics and sub-topics during stage 1) versus specific messages (i.e. SMS during stage 3). In other words, adolescents are better able to understand more specific, versus more general, information. For example, young participants in stage 1 rated alcohol and drugs as topics of very low interest (0.2 out of 3.0). However, adolescent participants in stage 3 rated the SMS in the sub-domain on alcohol, tobacco and drugs as of high interest (3.5 out of 4.0). Our team found similar results in an earlier study in Peru, where it was difficult for adolescent participants to think about abstract concepts, in this case, topics that influence their sexuality. At a later timepoint in the same study, the same adolescents were able to think about the same concepts more easily when these concepts were made more concrete, by putting them into role plays that the adolescents themselves acted out [28]. The results of the current study mirror what happened in the previous study. When concepts were abstract, as in the general topics and sub-topics, young participants seemed to be less able to understand and provide opinions about the topic. When concepts were much better defined, as in the SMS, young people were better able to understand and provide opinions and feedback.

Broader societal beliefs in Peru related to sexuality and gender may have also influenced what participants wanted from their messaging. Participants perceived that females should only be informed about female sexuality and males about male sexuality and that the sexuality of young women should be “hidden.” Participants also conceived of masturbation as a “normal” expression of sexuality for males, but not for females. A study on gender in adolescents in the neighboring country of Chile had similar findings: it was expected or accepted that females had to project an image of ignorance in relation to their sexuality and masturbation was conceived as a natural part of sexual development only for males [33]. These conceptions align with the conservative culture of Peru, where the influence of the Catholic Church is long-standing and influences many people’s beliefs regarding SRH [34]. Pressure from conservative social groups in the country has resulted in the Ministry of Education (MINEDU) recently weakening components of the National Basic Sexuality Education Curriculum that discussed gender norms and gender equality [35]. The perspectives shared by young people in this study reinforce why it is important that comprehensive sexuality education include gender norms and gender equality from an early age [36]. It also highlights unexpected tension that may arise during a participatory content development process that strives to consider the preferences of young people, while still promoting equity and positive social norms. Ultimately, for the ARMADILLO content, no messages were censored based on the above concerns.

ARMADILLO’s activities also provided insight into aspects of adolescent-related and youth-related health and wellbeing where more attention is needed. One important finding relates to the presence of violence in the lives of the adolescents and youth in this context. In the community consultations in stage 1, participants spontaneously mentioned intimate partner violence as an important topic and individual sharing during those community consultations also revealed household violence and sexual abuse of adolescents, including at the hands of close relatives. In the focus groups in stage 3, text messages about violence received high scores, which affirms the demand for information on the subject. Our results affirm the findings of past studies and reports in Peru, which have found high rates of violence in the country [37–41]. A study in Peru found the following for cases of sexual abuse: in 74% of cases, the perpetrators were part of the victim’s close environment; and in 38% of cases, the perpetrators were consanguineous family members [42]. Unfortunately, violence against women, children and adolescents in Peru is perpetuated generationally and is reproduced by its cultural acceptance [43], weak reporting mechanisms, lack of information regarding rights, and the population’s distrust of providers and institutions that should offer protection [38, 44]. Taking into account the distrust of potential protectors and that it is often family members who practice or cover up violence, affected adolescents are left extremely vulnerable [45]. These findings affirm the importance of including information about violence in the ARMADILLO platform, to disseminate information about what constitutes abuse and violence; to reinforce information about each individual’s rights and value as a person; and to provide information about available services and build trust towards these services.

Additionally, this study reinforces previous research showing that Peruvian young people lack awareness of and access to information about the range of contraceptive methods and in particular the most effective methods. For instance, most participants reported being aware of only emergency contraceptive pills, but not of regular contraceptive pills. Participants also described misconceptions regarding significant side effects of several contraceptive methods. Our findings affirm results from studies with young people in other LMICs, that youth hold several negative beliefs about contraception, especially LARCs [46–48]. WHO recommendations for preventing early pregnancies call for increasing the use of contraceptives among adolescents who are sexually active and do not want to get pregnant [49]. A recent systematic review of interventions to prevent adolescent pregnancy in LMICs found that the provision of contraception was highly effective and even led to declines in adolescent pregnancy rates [50]. However, in a recent survey of 2528 adolescents in different regions of Peru about what they learn during sex education in school, contraception was one of the least discussed topics and only 5 in 10 participants reported learning about where they could access contraception or how to use contraceptive methods [51]. Our results, in the context of current Peruvian policy and practice related to adolescent SRH, demonstrate the importance of including comprehensive, medically-accurate information about contraceptive methods and how to access and use these methods as part of the ARMADILLO system.

Our work has some limitations. Regarding the community consultations, it is possible that some SRH topics of interest to adolescents and youth may have not been verbalized due to the presence of adults. We sought to minimize this limitation by openly prioritizing the participation of youth over that of adults (who were present primarily as observers) and by providing a confidential option for adolescents and youth to also individually – versus publicly – share topics of interest. Regarding the focus groups, we may have received different results if we had divided the adolescents and youth by sex. However, we wanted to promote as much dialogue as possible and therefore included males and females in the same groups. We offered individual opportunities for contributions to try to address any limits to sharing this may have occasioned. One final limitation is that stage 1 and stage 3 activities were not audio recorded, as the primary aim of this formative phase was to determine the topics to include in the SMS system, develop and refine a library of SMS content and to achieve this goal through individual- and group-based participant sharing and real-time review, refinement and consensus of the ideas shared in groups. However, the team’s extensive notes and a debriefing among the team (using the notes and participants’ written material) after each activity session ensured a detailed review of the information shared.

## Conclusions

The results provide valuable information for the participatory design of SRH interventions for young people and strongly affirm the value of including adolescent and youth in the entire intervention-building process. This study confirms the importance of addressing the topics that are of greatest interest and importance to young people. It also provides critical information about what adolescent and youth know and how they think, enabling us to understand their perspective and literally speak their language, all of which was integrated into the resulting SMS system. Finally, the results also provide future directions for programmatic, research and policy efforts with young people in Peru (and Latin America), in particular around gender norms, interpersonal violence, and access to SRH information and services.

## Supplementary information

**Additional file 1.** Desarrollo de una intervención con mensajes de texto (SMS) sobre salud sexual y reproductiva con adolescentes y jóvenes en Perú

## Data Availability

Not applicable.

## Abbreviations

ARMADILLO: Adolescent/Youth Reproductive Mobile Access and Delivery Initiative for Love and Life Outcomes
ASK: Access, Service & Knowledge
DHS: Demographic and Health Survey
HIV: Human Immunodeficiency Virus
HIV/AIDS: Human Immunodeficiency Virus / A*cquired immune deficiency syndrome*
HRP: UNDP/UNFPA/UNICEF/WHO/World Bank Special Programme of Research, Development and Research Training in Human Reproduction
ICRH-K: International Centre for Reproductive Health Kenya
IUD: IntraUterine Device
LARCs: Long-Acting Reversible Contraceptives
LMICs: Low- and middle-income countries
MINEDU: Ministerio de Educacion del Peru
MINSA: Ministerio de Salud del Peru
MOH: Ministry of Health in Peru
SMS: Short message service
SRH: Sexual and Reproductive Health
STIs: Sexual transmitted infections
TPH: Tropical and Public Health
UPCH: Universidad Peruana Cayetano Heredia
WHO: World Health Organization
