# Supplementary material for: Developing an SMS text message intervention on sexual and reproductive health with adolescents and youth in Peru
Source: Reprod Health. 2020 Jul 31;17:116. doi: 10.1186/s12978-020-00943-6 (PMC7393715; doi:10.1186/s12978-020-00943-6)
Supplement: Supplementary file 1 — Additional file 1. Desarrollo de una intervención con mensajes de texto (SMS) sobre salud sexual y reproductiva con adolescentes y jóvenes en Perú [file 12978_2020_943_MOESM1_ESM.docx]

**Desarrollo de una intervención con mensajes de texto (SMS) sobre salud sexual y reproductiva con adolescentes y jóvenes en Perú**

Fiorella Guerrero,^1^ Nora Lucar,^1^ Mijail Garvich Claux, ^1^ Marina Chiappe, ^1^ Jose Perez-Lu, ^1^ Michelle J Hindin,^2^ Lianne Gonsalves,^345^ Angela M Bayer^1^

1. Facultad de Salud Pública y Administración, Universidad Peruana Cayetano Heredia, Lima, Perú

2. Reproductive Health Program, Population Council, Nueva York, Estados Unidos

3. Departamento de Salud Reproductiva e Investigaciones Conexas, incluye el Programa Especial PNUD / UNFPA / UNICEF / OMS / Banco Mundial de Investigaciones, Desarrollo y Formación de Investigadores sobre Reproducción Humana de la Organización Mundial de la Salud, Ginebra, Suiza.

4. Swiss Tropical and Public Health Institute (Swiss TPH), Basilea, Suiza

5. Basel University, Basilea, Suiza

**Resumen**

**Antecedentes**

El acceso mejorado a servicios e información sobre salud sexual y reproductiva (SSR) es esencial para apoyar a los adolescentes y jóvenes en la toma de decisiones informadas, así como para optimizar los resultados de cada joven sobre su SSR, su salud y bienestar, y sobre el desarrollo social y económico presente y futuro de los países. Para ello, los teléfonos móviles ofrecen oportunidades para que los jóvenes accedan de manera privada a contenidos sobre SSR y puedan ser derivados a servicios de SSR. El objetivo de este estudio fue desarrollar los contenidos para una plataforma de SMS (servicio de mensajes cortos o "mensaje de texto") junto con adolescentes y jóvenes de tres regiones del Perú (Lima, Ayacucho y Loreto) como parte del estudio ARMADILLO (por sus siglas en inglés de “Uso de mensajes de texto a celulares para mejorar resultados de amor y vida en adolescentes y jóvenes”).

**Método**

El desarrollo del contenido se desarrolló en tres etapas. Durante la Etapa 1, realizamos consultas comunitarias con adolescentes de entre 13 y 17 años, jóvenes de entre 18 y 24 años y profesionales que trabajan con jóvenes a través de los sectores de educación y salud ("asesores adultos") a fin de identificar y calificar temas de interés de SSR a través de actividades como lluvias de ideas, libres y guiadas, y de una actividad individual para compartir ideas por escrito. Durante la Etapa 2, el equipo identificó los dominios, subdominios y contenidos preliminares para la plataforma de SMSs. Durante la Etapa 3, realizamos grupos focales con adolescentes para validar el contenido de los SMSs, incluyendo una calificación individual y una retroalimentación grupal para cada uno de los SMS. En las retroalimentaciones grupales, les preguntamos sobre sus impresiones y comprensión general, así como sus opiniones sobre el lenguaje y la utilidad de los SMS.

**Resultados**

Participaron un total de 172 adolescentes y jóvenes de entre 13 y 24 años, y 20 asesores adultos. Los adolescentes y jóvenes participaron de lluvias de ideas donde identificaron y calificaron los temas y subtemas de SSR que condujeron a la estructura inicial de la plataforma de SMSs, con 9 dominios, 25 subdominios y 146 borradores de SMSs. Los adolescentes calificaron con puntajes altos a los SMS, donde todos los subdominios recibieron puntajes promedio de 3.0 o más (sobre 4.0) para todos los SMS incluidos. Los adolescentes también proporcionaron sugerencias para optimizar los contenidos, incluyendo mejoras en la claridad de los mensajes, lo que resultó en nuevos SMS con contenidos amigables para adolescentes en un lenguaje simple y directo. Este proceso también reveló que los adolescentes carecían de conocimientos y tenían conceptos erróneos relacionados a métodos anticonceptivos.

**Conclusión**

Este estudio detalla el proceso sistemático utilizado para el desarrollo de información de SSR relevante y accesible mediante un enfoque participativo. Documentamos información crítica sobre lo que los jóvenes saben y cómo piensan, lo que nos permitió comprender su perspectiva y, literalmente, hablar su propio idioma. Los resultados también proporcionan ideas sobre lineamientosfuturos para los esfuerzos programáticos, de investigación y de políticas con jóvenes, en particular aquellos en torno a normas de género, violencia interpersonal, acceso a la información y servicios de SSR, en entornos similares.

**Palabras clave**

# Adolescentes, jóvenes, salud sexual y reproductiva, enfoque participativo, grupos focales, mensajes de texto, Perú

# Resumen en lenguaje sencillo

# Mejorar los resultados de salud sexual y reproductiva (SSR) de los jóvenes requiere de la difusión de información sobre temas de SSR para los jóvenes a fin de brindarles las herramientas y el apoyo necesario para la toma de decisiones informadas. La difusión de información a través de mensajes de texto o SMS es una estrategia prometedora debido a la privacidad de los SMS y la amplia disponibilidad de teléfonos celulares entre adolescentes y jóvenes.

# En este estudio, describimos cómo trabajamos con adolescentes y jóvenes peruanos para desarrollar la estructura y el contenido de una intervención piloto para enviar SMS sobre SSR a jóvenes en Perú. Este estudio se realizó en tres regiones del Perú (Lima, Ayacucho y Loreto). Iniciamos con consultas comunitarias, donde los adolescentes, jóvenes y asesores adultos intercambiaron ideas sobre temas de interés en SSR para los jóvenes. En una segunda etapa, el equipo desarrolló una estructura inicial para la plataforma de SMS así como SMS preliminares. En la tercera etapa, organizamos grupos focales para pedir a los adolescentes opiniones sobre cada uno de los SMS. De esta forma, los jóvenes participaron activamente durante todo el proceso.

# Nuestros hallazgos muestran lo que los jóvenes saben y cómo piensan acerca de los temas de SSR, lo que nos permitió comprender su perspectiva y, literalmente, hablar en su mismo idioma para proporcionarles la información que necesitan y que recibirán, aquello que considerarán accesible y útil, y finalmente, aquello que tomarán en cuenta para poder tomar buenas decisiones. Los resultados también proporcionan lineamientos futuros para los esfuerzos programáticos, de investigación y de políticas públicas con jóvenes, especialmente en torno a normas de género, violencia interpersonal y acceso a información y servicios de SSR en entornos similares.

# Introducción

A nivel mundial, hay aproximadamente 1.9 billones de adolescentes y jóvenes de entre 10 y 24 años, que representan aproximadamente un cuarto de la población total (1). En Perú, hay aproximadamente 8,7 millones de adolescentes y jóvenes de entre 10 y 24 años que representan el 28% de la población total (2). Teniendo en cuenta el significativo tamaño de esta población, es fundamental invertir en ellos para la mejora de sus conocimientos y habilidades, incluidos aquellos relacionados a la salud sexual y reproductiva (SSR), a fin de optimizar tanto el desarrollo de cada joven, sus resultados en SSR y otros resultados relacionados a su salud y bienestar, incluyendo el desarrollo social y económico presente y futuro de Perú.

Aunque la prevalencia del embarazo adolescente ha disminuido en todo el mundo, en Perú, el embarazo adolescente ha permanecido sin cambios en el tiempo y continúa siendo algo inesperado para la mayoría de las adolescentes. Según la Encuesta Demográfica y de Salud del Perú de 2017 (DHS / ENDES), el 13,4% de las mujeres de 15 a 19 años estaban embarazadas o criando un niño (3), un porcentaje que no ha cambiado significativamente desde 1991/92 (4). La tasa de embarazos es mayor entre las adolescentes que viven en zonas rurales versus la de aquellas en zonas urbanas (23% frente a 11%), y mayor para las que viven en la selva (23%) versus aquellas que viven en la sierra y en la costa (13% y 12%). Además, el porcentaje de embarazo es más alto entre quienes tienen solo educación primaria comparado con quienes tienen educación secundaria (45% vs. 12%), y para aquellas en el quintil económico más pobre comparado con aquellas en el más rico (24% vs. 4%). En 2016, el 66% de las peruanas de entre 15 y 19 años que habían estado embarazadas informaron que su último embarazo fue inesperado (58% no planificado y 8% no deseado). Los reportes de embarazos inesperados han aumentado constantemente en el tiempo, pasando del 47% en 1991/2 al 55% en 2000 (5). Esto contrasta con el grupo de mujeres de entre 20 y24 años, entre las cuales la tasa de fecundidad específica por edad ha disminuido de 200 nacimientos por 1,000 mujeres de 20 a 24 años en 1991/92 a 112 por 1,000 en 2015/16 (6).

Las infecciones de transmisión sexual (ITS), incluido el VIH, son otro tema de SSR que afecta a los jóvenes peruanos. Un gran estudio poblacional con jóvenes de 18 a 29 años de edad en ciudades peruanas más pequeñas, sin incluir la capital, Lima, arrojó las siguientes estimaciones de prevalencia: 6,5% en mujeres y 4,2% en hombres para clamidia; 4,9% en mujeres y 0,3% en hombres para tricomonas; 0,4% en mujeres y 0,5% en hombres para sífilis; 0,1% en mujeres y hombres para gonorrea; y 0,1% en mujeres y 0,5% en hombres para VIH (7).

Aunque Perú ofrece servicios de salud “amigables para adolescentes”, incluidos servicios de SSR, estos no son tan amigables para adolescentes como se propondría. El Ministerio de Salud del Perú (MINSA) ha trabajado para crear y expandir los servicios de salud amigables para adolescentes, aumentando de 213 establecimientos de salud en 2006 a más de 2,800 establecimientos en 2016. MINSA declara que estos servicios ofrecen horarios de atención, proveedores y servicios amigables para adolescentes, incluidos servicios de SSR (8). Sin embargo, un reciente informe de la Defensoría del Pueblo del Perú encontró que estos servicios de salud amigables para adolescentes en realidad no se adaptaron a las necesidades de los adolescentes (9). Además, los servicios exigían también el acompañamiento de los padres, algo contrario a la legislación vigente (9). Una cuarta parte (25%) de los establecimientos de salud requerían el acompañamiento de los padres o tutores para que los menores accedieran a los servicios, a pesar de que la Norma Técnica de Salud de Planificación Familiar ya no lo requiere desde agosto de 2017 (10).

En vista de los desafíos mencionados que enfrentan los adolescentes para acceder a nformación y servicios de SSR necesarios, es fundamental desarrollar estrategias innovadoras para mejorar el acceso de los adolescentes a información relevante y confiable a través de canales fuera de los servicios de salud. Uno de esos canales son los teléfonos móviles, a través de los cuales los adolescentes pueden acceder fácilmente a información sobre diferentes temas de SSR, incluida información sobre SSR y otros servicios disponibles a través del sistema de salud pública y otros sistemas. La alta penetración de teléfonos móviles a nivel mundial, incluso entre adolescentes y jóvenes, confirma el potencial de este canal alternativo. Por ejemplo, entre peruanos de 15 a 29 años, la posesión teléfonos móviles es mayor que en la población general, donde el 71% de los jóvenes peruanos informa tener uno (11).

Se han realizado varias intervenciones para proporcionar información sobre SSR a los jóvenes en diferentes países de bajos y medianos ingresos (PBMI) que utilizan teléfonos móviles. Por ejemplo, el programa mCenas! utilizó un sistema interactivo de SMS (servicio de mensajes cortos o "mensaje de texto") para proporcionar mensajes narrativos e informativos a personas de 15 a 24 años en Mozambique para ampliar sus conocimientos sobre métodos anticonceptivos, y tratar los mitos relacionados a ellos y las barreras de acceso más comunes (12). El programa “Aprendiendo de la Vida” (Learning about Living en inglés) utilizó un sitio web para proporcionar educación preventiva sobre salud materna, VIH / SIDA y violencia de género a personas de 10 a 21 años en Nigeria (13). La iniciativa de “Acceso, Servicio y Conocimiento” (Access, Service and Knowledge or ASK en inglés) utilizó plataformas móviles y electrónicas para difundir información sobre SSR y VIH / SIDA a personas de 10 a 24 años en cinco países en África y dos en Asia (14). YoungAfricaLive utilizó una plataforma móvil comunitaria para proporcionar información sobre amor, sexo y relaciones a los jóvenes en Kenia, Sudáfrica y Tanzania, teniendo en cuenta su situación social, cultural y económica (15). Estos estudios demostraron aumentar los conocimientos sobre anticonceptivos (16) y mejorar los conocimientos y actitudes hacia indicadores de sexualidad como el VIH / SIDA y la violencia de género (13) entre los jóvenes participantes. Sin embargo, existe una limitada información disponible sobre las fases formativas y muchos de los programas no han logrado incorporar las perspectivas de los jóvenes en el desarrollo del contenido de los programas, a pesar de las pautas internacionales sobre cómo la integración de las voces de los jóvenes puede aumentar la efectividad de un programa (17). Del mismo modo, pocas de estas intervenciones se han evaluado rigurosamente y la evidencia sobre la viabilidad y efectividad de las intervenciones en SSR basadas en teléfonos móviles dirigidas a adolescentes y jóvenes es limitada.

# Para abordar estas brechas de investigación, la Organización Mundial de la Salud (OMS), la Universidad Peruana Cayetano Heredia (UPCH) y el Centro Internacional de Salud Reproductiva de Kenia (ICRH-K, siglas en inglés) iniciaron "ARMADILLO" (Uso de mensajes de texto a celulares para mejorar resultados de amor y vida en adolescentes y jóvenes) con el objetivo general de generar evidencia sobre el efecto que tiene el acceso a la información de SSR mediante los SMS sobre los resultados relacionados con la salud entre adolescentes. Las intervenciones de mHealth dirigidas a adolescentes y jóvenes deben desarrollarse y probarse con ellos a fin de identificar e incorporar contenidos de información sobre SSR y diseños de sistema apropiados (18,19), y documentar estrategias exitosas para involucrarlos significativamente. El objetivo específico de la primera fase de ARMADILLO fue desarrollar la plataforma de SMS (mensaje de texto) ARMADILLO, incluidos los temas y subtemas de SSR, el diseño del sistema de SMS y el contenido de los SMS, utilizando un enfoque participativo con los jóvenes. Este manuscrito documenta el proceso participativo utilizado en Perú, que consistió en un esfuerzo en tres etapas para desarrollar la plataforma en colaboración con adolescentes y jóvenes de 13 a 24 años. El proceso y los resultados podrían ser útiles para informar otras iniciativas, especialmente en los países de América Latina, que tienen como objetivo abordar los desafíos en SSR que enfrentan los adolescentes y los jóvenes.

# Método

El desarrollo del contenido de ARMADILLO incluyó tres etapas para desarrollar la plataforma de SMS junto con adolescentes y jóvenes: (1) trabajar con adolescentes y jóvenes para identificar y calificar los temas que son de mayor interés para ellos y otros adolescentes y jóvenes de su edad; (2) desarrollar la estructura inicial de la plataforma de SMS, incluidos los temas, subtemas y los SMS; y (3) validar los SMS preliminares con adolescentes. (Ver (20) para más detalles sobre ARMADILLO.)

**Contexto de estudio**

La investigación formativa para ARMADILLO se llevó a cabo en tres lugares de Perú, un país con una población estimada de 32 millones (21). El estudio se implementó en distritos urbanos y periurbanos de las tres regiones naturales del país: Lima, la capital, ubicada en la costa; Ayacucho, en la sierra; y Loreto, en la selva. Estas regiones también fueron seleccionadas en base a sus altas tasas de embarazo adolescente: Lima Metropolitana, 8,3%; Ayacucho, 13,8%; y Loreto, 25,6% (22).

**Recopilación de datos**

Las tres etapas del estudio se llevaron a cabo entre noviembre de 2015 y julio de 2016.

***Etapa 1. Consultas comunitarias con adolescentes, jóvenes y profesionales para la identificación de temas de interés para jóvenes.***

La primera etapa en el desarrollo de mensajes consistió en consultas informales y comunitarias. Estas incluyeron participantes adolescentes y jóvenes, así como "asesores adultos" profesionales, en las treslocalidades. El equipo de estudio contactó a varios adultos en cada comunidad ("guardianes") que habían trabajado ampliamente con adolescentes y jóvenes. Estos guardianes invitaron a jóvenes de las escuelas, de establecimientos de salud y de la comunidad en general a participar, a fin de obtener una muestra diversa de participantes que incluyera adolescentes dentro y fuera de la escuela, jóvenes que trabajaban y no trabajaban, y algunos jóvenes a la espera del nacimiento de su hijo y/o padres jóvenes. Los asesores adultos eraon adultos que se relacionaban estrechamente con jóvenes en su trabajo profesional, incluidos profesionales de la educación, como funcionarios escolares y maestros, profesionales de la salud, como obstetras y psicólogos, y líderes comunitarios. Los guardianes también reclutaron asesores adultos que sirvieron principalmente como observadores.

Llevamos a cabo seis consultas comunitarias en los tres sitios de estudio, con dos reuniones por cada lugar, con la siguiente estructura: una reunión con mujeres y hombres de entre 13 y 17 años y un asesor adulto; y una reunión con mujeres y hombres de entre 18 y 24 años y un asesor adulto. Se priorizaron las ideas y perspectivas de los jóvenes, y los adultos contribuyeron con sus ideas solo después de que los adolescentes y jóvenes habían terminado de opinar. Cada consulta comunitaria fue facilitada por AMB y por un segundo miembro del equipo. Las reuniones se llevaron a cabo en español y duraron entre 1,5 a 2 horas.

Las consultas comunitarias fueron diseñadas para generar y priorizar las áreas temáticas de SSR. Primero, a los participantes se les dio la siguiente indicación para generar la lluvia de ideas: "Pensando en salud sexual y reproductiva, ¿cuáles son los temas o las cosas que son de mayor interés para ustedes y otros adolescentes y jóvenes que conocen?" Luego de que no surgieron nuevas ideas, el facilitador inició una lluvia de ideas guiada, mencionando varios temas de SSR que no habían sido mencionados por los participantes en la lluvia de ideas libre y que se habían incluido en otros proyectos y estudios de SSR similares con jóvenes en otros contextos (23–27) y en Perú (28–30). Por cada tema mencionado, los participantes trabajaron hasta llegar a un consenso sobre si el tema era " de no interés", "de interés" o "de gran interés" para ellos. Los participantes seleccionaron a alguien para tomar notas, quien escribió todas las respuestas en un papel borrador, las cuales se registraron sin atribución a ningún participante en específico. Finalmente, los participantes recibieron una hoja de papel para escribir cualquier otro tema de SSR que pudiera ser de su interés para facilitar la expresión privada de ideas que podrían haberse omitido durante las actividades grupales debido a vergüenza, miedo a ser juzgado u otras razones. Estos temas se agregaron a la lista de la lluvia de ideas libre en el papel borrador. Además de las notas en el papel borrador y las notas individuales de los participantes, el equipo también tomó notas detalladas sobre todas las conversaciones durante las consultas comunitarias. Ninguna de las notas registró información de identificación sobre los participantes.

***Etapa 2. Desarrollo de la estructura inicial de la plataforma de SMS y del contenido preliminar de los SMS por parte del equipo***

La estructura inicial de la plataforma se desarrolló en dos pasos. Primero, cuatro miembros del equipo revisaron por separado los temas y las puntuaciones de la priorización de la etapa 1 para crear una posible estructura para la plataforma, que incluyó dominios (temas) y subdominios (subtemas). En segundo lugar, los miembros del equipo se unieron para compartir sus ideas y crear una propuesta conjunta de dominios y subdominios. Posteriormente, el equipo desarrolló los SMS, creando de 2 a 10 SMS por cada subdominio de la plataforma. El equipo revisó cuidadosamente toda la documentación de las consultas comunitarias en la etapa 1, incluyendo los temas de interés y el lenguaje que los participantes adolescentes y jóvenes utilizaron al hablar sobre estos temas, para redactar el contenido de los SMS. Los SMS podrían contener hasta 140 caracteres, debido a las características de los teléfonos móviles peruanos. Se desarrollaron un total de 146 SMS.

***Etapa 3. Grupos focales con adolescentes para la revisión y validación de los SMS propuestos.***

Para validar el contenido de los SMS, seguimos un muestreo propositivo (con asistencia cercana de los guardianes adultos descritos en la etapa 1) para reclutar adolescentes de los tres sitios de estudio para participar en 12 grupos focales. Realizamos cuatro grupos focales en cada sitio con la siguiente estructura: dos grupos con mujeres y hombres de 13 a 15 años; y dos grupos con mujeres y hombres de 16-17 años. Incluimos solo a jóvenes de 13-17 años en esta etapa en respuesta al pedido de los actores claves nacionales de ver un enfoque especial en el período de adolescencia, dadas las estadísticas de embarazo adolescente e inicio sexual temprano en Perú. Los participantes en la etapa 3 no participaron en la etapa 1.

Para los grupos focales, cada grupo de participantes evaluó de 36 a 37 SMS (146 SMS en total) y todos los SMS fueron revisados ​​por un grupo focal en cada lugar de estudio. En cada grupo focal, cada SMS fue revisado uno por uno, con una evaluación individual seguida de una evaluación grupal del SMS. Para la evaluación individual, cada adolescente recibió un formulario para calificar cada SMS. La evaluación individual consistió en una sola pregunta, "¿Qué opinas de este mensaje de texto?", con opciones de respuesta en escala de Likert acompañadas de caras tristes y sonrientes en cuatro puntos que van desde muy malo (1 punto) a muy bueno (4 puntos). Para la evaluación grupal, cada SMS se proyectó en un muro/pantalla y se solicitaron opiniones grupales. El facilitador generó un diálogo al preguntar sobre: ​​la impresión general del mensaje de los participantes ("¿Qué opinas de este mensaje? Si te llegara este mensaje, ¿qué te parecería?"); la comprensión del mensaje ("Si tuvieses que explicar lo que dice el mensaje, ¿qué dirías?"); el lenguaje del mensaje ("¿Se entiende el lenguaje del mensaje? ¿Usa las palabras adecuadas?"); y la utilidad del mensaje para los participantes ("¿Cómo se puede mejorar este mensaje? ¿Hay algo que no les guste del mensaje o que creen que a los jóvenes que conocen no les gustaría?"). Los grupos focales fueron facilitados por un joven investigador con experiencia en ciencias sociales e investigación cualitativa con jóvenes en Perú. Las reuniones se llevaron a cabo en español y duraron entre 1,5 y 2 horas. Un segundo miembro del equipo tomó notas detalladas de todos los comentarios para cada uno de los SMS para su posterior análisis y la incorporación de los comentarios de los jóvenes en cada SMS.

***Gestión y Análisis de Datos***

Todos los análisis y la gestión de datos se realizaron en Microsoft Excel (Microsoft Corp., Redmond, WA, EE. UU.). Para la etapa 1, creamos una lista maestra de todos los temas mencionados durante las consultas comunitarias y les asignamos un puntaje entre 0 y 3 a cada tema, de acuerdo con las opiniones de los participantes: 3 para los temas que los participantes mencionaron espontáneamente durante la lluvia de ideas libre o al compartir individual; 2 para los temas que los participantes calificaron como "de gran interés" durante la lluvia de ideas guiada; 1 para temas que los participantes calificaron como "de interés" durante la lluvia de ideas guiada; y 0 para temas que los participantes no mencionaron o que los participantes dijeron que no eran "de interés" para ellos durante la lluvia de ideas guiada. Los subgrupos de participantes se establecieron en función de la edad junto con la localidad (por ejemplo, adolescentes de 13-17 años de Lima; jóvenes de 18-24 años de Yurimaguas). Se creó una columna para cada subgrupo de participantes. Se generaron puntuaciones por cada subdominio y por cada dominio, que es la media de todos los subdominios dentro de ese dominio, utilizando los subdominios y dominios desarrollados durante la etapa 2. Estas puntuaciones se desarrollaron para cada subgrupo de participantes, y para los grupos de 13-17 y 18-24 años en general.

Para la etapa 3, calculamos el puntaje promedio para cada SMS, para cada localidad y en general. También calculamos el puntaje promedio para cada dominio y subdominio de la plataforma. Todos los comentarios de los participantes con respecto a cada SMS también se consolidaron en Excel. Todos los comentarios para cada SMS se analizaron para sintetizar los mensajes principales e integrarlos en el contenido final y la redacción de los SMS. Dos miembros del equipo analizaron de forma independiente todos los comentarios de los participantes, hicieron un resumen de los comentarios principales y redactaron SMS revisados. Luego, todo el equipo se reunió para: discutir los comentarios principales; volver a revisar los comentarios de los mismos participantes cuando hubo discrepancias entre los dos revisores; revisar las modificaciones de SMS propuestas; y desarrollar una versión final de los SMS.

**Consideraciones éticas**

Todos los participantes adolescentes y jóvenes dieron su asentimiento o consentimiento informado por escrito (dependiendo de si tenían más de 18 años) antes de participar en las consultas comunitarias o en las actividades de investigación como los grupos focales de discusión. Los padres y tutores de los participantes menores de 18 años también dieron su consentimiento informado por escrito para la participación de su hijo. Si los participantes revelaron casos de haber experimentado o presenciado violencia o manifestado angustia mental durante cualquier actividad, se les proporcionó información sobre los servicios de salud general, mental y de violencia en sus comunidades, incluidos los servicios que son específicamente para jóvenes. El estudio ARMADILLO fue aprobado por el Comité de Revisión de Ética de Investigación de la Organización Mundial de la Salud y el Comité de Ética de la Universidad Peruana Cayetano Heredia.

**Resultados**

***Etapa 1. Consultas comunitarias con adolescentes, jóvenes y profesionales para identificar temas de interés para los jóvenes.***

Un total de 68 participantes de 13 a 24 años fueron parte de las seis consultas comunitarias (ver Tabla 1). En las tres consultas con adolescentes de 13 a 17 años, el 69% fueron mujeres y el 31% hombres. En las tres consultas con jóvenes de 18 a 24 años, el 64% fueron mujeres y el 36% hombres. En cada grupo hubo participantes jóvenes y adolescentes que ya eran padres o que estaban esperando un hijo. También participaron veinte adultos: Lima (3); Yurimaguas (5); y Ayacucho (12).

**Tabla 1: Participantes adolescentes y jóvenes en consultas comunitarias de la Etapa 1, por grupo de edad y sexo, Lima, Yurimaguas and Ayacucho, Perú, 2015**

|  | **Jóvenes de 13-17 años** | | **Jóvenes de 18-24 años** | |
| --- | --- | --- | --- | --- |
|  | **Mujeres** | **Hombres** | **Mujeres** | **Hombres** |
| **Lima** | 7 | 6 | 4 | 7 |
| **Yurimaguas** | 4 | 3 | 4 | 5 |
| **Ayacucho** | 11 | 1 | 15 | 1 |
| **TOTAL** | 22 | 10 | 23 | 13 |

Los participantes adolescentes y jóvenes mencionaron muchos temas de interés, que luego se agruparon en temas generales y subtemas más específicos de SSR. Los adolescentes mostraron una falta de conocimientos sobre las diferentes terminologías de SSR y, en particular, sobre los métodos anticonceptivos. Por ejemplo, solo sabían de los anticonceptivos orales de emergencia, también conocidas como la píldora del "día siguiente", que se usan en situaciones de emergencia o imprevistos, pero no tenían conocimientos sobre las píldoras anticonceptivas hormonales regulares que se usan como contracepción regular. Tampoco conocían los métodos anticonceptivos reversibles de larga duración (ARLD), como los dispositivos intrauterinos (DIU) y los implantes, ni sus beneficios sobre otros métodos anticonceptivos. Una vez informados durante las lluvias de ideas guiadas, los participantes expresaron su temor sobre los posibles efectos secundarios negativos de los métodos anticonceptivos regulares y particularmente los ARLD, incluida la esterilidad, ya que los consideraron inapropiados para adolescentes. Incluso el uso del condón no se entendía bien, ya que era percibido como el mejor método para prevenir el embarazo, pero no como el mejor método para prevenir las ITS.

Los diez temas de SSR y los subtemas respectivos que resultaron de las consultas con la comunidad se muestran en la Tabla 2, presentada de acuerdo con la calificación que les asignaron los participantes adolescentes y jóvenes. Los temas mejor calificados fueron identidad, ITS, métodos de barrera y anticonceptivos específicos. Las categorías con menor puntaje fueron alcohol y drogas, derechos y políticas públicas de SSR y embarazo. El puntaje promedio varió entre 0,2 a 1,7 sobre un posible 3,0. Una de las principales razones en los puntajes más bajos fue la falta de interés en ciertos subdominios. Por ejemplo, la 'violencia' como tema tuvo un puntaje promedio de 1,2 porque los participantes indicaron poco interés en los subdominios de abuso sexual y violación (puntajes de 0,4), pero hubo un mayor interés en el subdominio de violencia de pareja (puntaje de 2,0).

El interés en el tema de SSR difería según la edad (ver Tabla 2). Los participantes adolescentes mostraron un mayor interés en la autoestima, la abstinencia y el VIH, y dieron la calificación más alta a la comunicación con sus familias, tanto en general como en temas relacionados con la sexualidad. Los participantes jóvenes de mayor edad mostraron un mayor interés en temas como la exploración de su sexualidad, síntomas de ITS y prevención, "amigos con derechos" (amigos que pueden participar en actividades sexuales sin considerarse "en una relación"), la presión de grupo para tener sexo y el acceso a servicios de salud, incluidos servicios psicológicos y de salud mental. Ambos grupos de edad estuvieron interesados ​​o muy interesados ​​en los métodos anticonceptivos (en general y en cada método específicamente), la violencia de pareja, el embarazo y el aborto.

Cuando los participantes tuvieron la oportunidad de escribir temas de interés, surgieron preguntas detalladas sobre dudas y curiosidades relacionadas con SSR, identidad y orientación sexual. Por ejemplo, "¿Por qué a algunas personas les gustan otras de su mismo sexo?", "Me gustaría saber más acerca de los hermafroditas", o "¿Es bueno o normal sentir amor por un hombre, si yo soy un hombre?". También surgieron preguntas sobre el abuso psicológico, físico y sexual en el entorno familiar, incluido "¿Cómo puedo hacer frente a la violencia y el alcoholismo de los padres?" o "¿Qué se puede decir a una adolescente que ha sido violada varias veces por su padrastro a los 6 años, que actualmente se siente despreciada por su madre y desea morir?".

**Etapa 2. Desarrollo de la estructura inicial de la plataforma SMS y del contenido preliminar de los SMSpor parte del equipo.**

El equipo desarrolló una estructura propuesta para la plataforma basada en un análisis profundo y crítico de la información compartida por los participantes en la etapa 1. El borrador final de la estructura de la plataforma se clasificó en nueve dominios o temas. (Ver detalles en la Figura 1.) Siete de estos dominios fueron "independientes", ya que todos los SMS relacionados con ese tema fueron incluidos en el dominio. Los dominios independientes fueron 1) ¿Quién soy? 2) ¿Quiénes me cuidan? 3) ¿Cómo disfruto? 4) ¿De qué me cuido? 5) ¿Cómo me cuido? 6) ¿Cómo me decido? y 7) Lo que todos callamos. El equipo decidió formular los dominios como preguntas, en lugar de como declaraciones, para tratar de atraer a los jóvenes y hacer que la plataforma "hable" a las preguntas que puedan tener. Los otros dos dominios fueron transversales, ya que los mensajes se referían a temas que podrían ser relevantes para muchos de los dominios independientes: 8) autoestima y motivación; y 9) acceso a los servicios del Ministerio de Salud. Además, los SMS incluidos en estos dominios transversales se distribuirían intercalados con cada uno de los dominios independientes.

La estructura inicial también incluyó 25 subdominios o subtemas, los cuales tenían entre 2 y 7 subdominios por cada dominio, dependiendo de la complejidad del dominio en sí. Cada subdominio tuvo entre 2 y 10 mensajes. El lenguaje de los mensajes fue redactado intencionalmente para ser dialogante, en lugar de autoritario, así como simple (al nivel de un adolescente) y coloquial, siguiendo el modelo de servicios basados ​​en SMS similares y populares que ya existían.

***Etapa 3. Grupos focales para revisar y validar los SMS con adolescentes***

Un total de 104 adolescentes participaron en los doce grupos focales en la etapa final. Esto incluyó seis grupos focales con 50 adolescentes de 13 a 15 años y seis grupos focales con 54 adolescentes de 16 a 17 años. De todos los participantes, el 52% fueron mujeres y el 48% hombres (ver Tabla 3).

**Tabla 3: Participantes en los grupos focales de la Etapa 3, por grupo de edad y sexo, Lima, Yurimaguas y Ayacucho, Perú, 2016**

|  | **13-15 años** | | **16-17 años** | |
| --- | --- | --- | --- | --- |
|  | **Mujeres** | **Hombres** | **Mujeres** | **Hombres** |
| **Lima** | 10 | 9 | 9 | 9 |
| **Yurimaguas** | 9 | 8 | 8 | 9 |
| **Ayacucho** | 9 | 5 | 9 | 10 |
| **TOTAL** | 28 | 22 | 26 | 28 |

*Resultados de la evaluación individual de los adolescentes para obtener impresiones generales de los SMS*

La Figura 1 presenta el puntaje promedio para todos los SMS incluidos en cada subdominio. En contraste con las calificaciones de los temas de SSR durante la etapa 1, muchas de las cuales fueron bajas, los SMS recibieron calificaciones uniformemente altas. Los subdominios mejor calificados fueron: la familia (3,7); abuso sexual (3,7); abstinencia (3,6); sexo transaccional (3,6); abstinencia secundaria (3,6); violencia (3,6); y amigos, pareja, alcohol, tabaco y drogas (todos con 3,5). Los subdominios restantes también recibieron altas calificaciones, de entre 3,0 y 3,4.

**Figura 1: Diseño de los dominios y subdominios para la plataforma de SMS ARMADILLO Perú y puntaje promedio para las calificaciones de los adolescentes de los mensajes SMS incluidos en cada subdominio, Lima, Yurimaguas y Ayacucho, Perú, 2016^1^**

*1 Los números en la Figura se basan en las calificaciones individuales de los adolescentes de los SMS durante los grupos focales de la Etapa 3. Los adolescentes calificaron cuánto les gustaba cada SMS, en una escala de 1 (muy malo) a 4 (muy bueno). Cada número representa la calificación promedio de todos los participantes para todos los SMS incluidos en un subdominio dado.*

# *Resultados de la evaluación grupal de los adolescentes para proporcionar sus perspectivas respecto a la facilidad de comprensión, el lenguaje y la utilidad de los SMS*

# Con respecto a la redacción, los participantes tuvieron diferentes tipos de sugerencias para mejorar la aceptabilidad y la comprensión de los mensajes. Estas sugerencias fueron similares en todos los grupos de edad. Una de ellas fue la sugerencia de adaptar los mensajes de acuerdo al sexo. Los adolescentes declararon que sentían que algunos mensajes eran inapropiados para mujeres o para hombres y que algunos mensajes deberían dirigirse solo al grupo al que se aplicaba el mensaje; por ejemplo, que las mujeres no deberían recibir información sobre la pubertad masculina y viceversa. Además, indicaron que algunos temas eran nuevos para ellos: por ejemplo, las adolescentes de ambos grupos de edad no estaban familiarizadas con el tema de la masturbación femenina. Por lo tanto, una sugerencia relacionada fue asegurarse de que los mensajes fueran muy específicos para que los adolescentes tuvieran una buena comprensión del contenido.

# Los participantes adolescentes también sugirieron refinar aún más el tono de ciertos mensajes y usar un lenguaje más familiar para captar la atención de los adolescentes. Por ejemplo, sugirieron cambiar el uso del imperativo (por ejemplo, "Haz esto para poder ...") a un lenguaje más neutral (por ejemplo, "Puedes hacer esto para poder ..."). También recomendaron el uso de palabras más usadas por los jóvenes, como "enamorado / enamorada" en lugar de "pareja íntima", "me gusta" en lugar de "preferencias", o "zona v" en lugar de "vagina". También recomendaron agregar frases como "no es broma" y signos de exclamación a mensajes específicos, para garantizar que los adolescentes crean y tomen en serio los contenidos.

# La Tabla 4 muestra una selección de mensajes de texto de ARMADILLO de diferentes dominios y subdominios en dos puntos diferentes durante este esfuerzo de investigación formativa: la versión preliminar de los SMS, creada por el equipo durante la etapa 2 basada en los resultados de la etapa 1; y la versión final de los SMS, después de integrar todos los comentarios que los adolescentes proporcionaron para cada SMS durante la etapa 3. Como se muestra en la Tabla, algunos mensajes cambiaron mínimamente mientras que otros cambiaron drásticamente. Para los SMS con cambios mínimos, los participantes a menudo querían agregar un mayor énfasis a cierto texto en el mensaje, por ejemplo, poniendo en mayúsculas la palabra "no" o agregando un signo de exclamación para darle énfasis adicional. Para los SMS con más cambios, los participantes querían modificar el idioma para hacerlo más amigable para los adolescentes o hacerlo más específico.

# Discusión

Este manuscrito es uno de los primeros en documentar procesos programáticos y de investigación participativos para construir sistemas de mensajes de texto para intervenciones de salud pública junto con adolescentes y jóvenes. La limitada evidencia existente ha demostrado que las intervenciones de cambio de comportamiento en SSR desarrolladas junto con participantes adolescentes y jóvenes son mejor recibidas (31,32). Por ejemplo, en un estudio en el Reino Unido que tuvo como objetivo reducir la incidencia de ITS a través de una intervención basada en SMS, los autores encontraron que para los mensajes que se probaron antes del despliegue inicial a través de discusiones grupales con jóvenes de entre 16 y 24 años mostraron que durante la implementación los mensajes probados se consideraban fáciles de entender y ninguno de ellos recibió una baja calificación (31).

Adoptamos un enfoque participativo similar para el proceso de desarrollo de la intervención de ARMADILLO. La investigación fue precedida por una etapa 1 de consulta, donde los participantes adolescentes y jóvenes decidieron los temas que querían incluir en la plataforma de SMS. Durante una etapa 2 preparatoria, el equipo desarrolló un sistema inicial basado completamente en dichas consultas comunitarias. Los mensajes que los participantes adolescentes evaluaron en la etapa 3, por lo tanto, fueron cuidadosamente elaborados en base a una extensa consulta inicial. Durante la recopilación de datos en los grupos focales, los participantes adolescentes dieron sus opiniones individuales y grupales sobre cada SMS potencial para la plataforma ARMADILLO. Los resultados del proceso participativo de ARMADILLO proporcionaron información clave sobre lo que los jóvenes peruanos prefieren, entienden y aceptan del contenido propuesto de una intervención digital de SSR. Además, también revelaron información, normas y comportamientos importantes relacionados con la salud donde se necesita más atención (digital o de otro tipo).

Los comentarios de los jóvenes reafirman lo que el trabajo previo con adolescentes halló: el lenguaje debería ser simple; permitir a los adolescentes relacionar los contenidos con experiencias personales; y tener un tono confiable, amigable y profesional (31). También muestran que la importancia y la relevancia se perciben de manera diferente para áreas temáticas amplias (por ejemplo, los temas y subtemas durante la etapa 1) frente a los mensajes más específicos (por ejemplo, los SMS durante la etapa 3). Nuestro estudio muestra que los adolescentes están en mejores condiciones de comprender información más específica, en comparación con información más general. Por ejemplo, los jóvenes participantes en la etapa 1 calificaron el alcohol y las drogas como temas de muy bajo interés (0,2 de 3,0). Sin embargo, los participantes adolescentes en la etapa 3 calificaron los SMS en el subdominio de alcohol, tabaco y drogas como de alto interés (3,5 de 4,0). Nuestro equipo encontró resultados similares en un estudio anterior en Perú, donde era difícil para los adolescentes participantes pensar en conceptos abstractos, en este caso, sobre temas que influyen en su sexualidad. En un momento posterior en el mismo estudio, los mismos adolescentes pudieron pensar en los mismos conceptos más fácilmente cuando estos conceptos se hicieron más concretos, poniéndolos en juegos de roles que los mismos adolescentes representaron (28). Los resultados del estudio actual reflejan lo que sucedió en el mencionado estudio anterior. Cuando los conceptos fueron abstractos, como en el caso de los temas y subtemas generales, los participantes jóvenes parecían ser menos capaces de comprender y proporcionar opiniones sobre el tema. Cuando los conceptos se definieron mucho mejor, como en los SMS, los jóvenes pudieron comprender y brindar sus opiniones y comentarios.

Las creencias sociales más extendidas en Perú relacionadas con la sexualidad y el género también podrían haber influido en lo que los participantes opinaron de los mensajes. Los participantes percibieron que las mujeres solo deberían ser informadas sobre la sexualidad femenina y los hombres sobre la sexualidad masculina y que la sexualidad de las mujeres jóvenes debería ser "ocultada". Los participantes también concibieron la masturbación como una expresión "normal" de la sexualidad para los hombres, pero no para las mujeres. Un estudio sobre género en adolescentes en el país vecino de Chile tuvo hallazgos similares: se esperaba o se aceptaba que las mujeres tuvieran que proyectar una imagen de ignorancia en relación con su sexualidad y la masturbación se concibió como una parte natural del desarrollo sexual solo para los hombres (33). Estas concepciones se alinean con la cultura conservadora de Perú, donde la influencia de la Iglesia Católica es histórica e influye en las creencias de muchas personas con respecto a la SSR (34). La presión de los grupos sociales conservadores en el país ha provocado que el Ministerio de Educación (MINEDU) haya reducido recientemente los componentes del Currículo Nacional de Educación Sexual Básica que discutía las normas y la igualdad de género (35). Las perspectivas compartidas por los jóvenes en este estudio refuerzan el porqué es importante que la educación integral en sexualidad incluya normas de género e igualdad de género desde una edad temprana (36). También destaca la tensión inesperada que puede surgir durante el proceso de desarrollo de contenido participativo, durante el cual se esfuerza por considerar las preferencias de los jóvenes y al mismo tiempo promover la equidad y las normas sociales positivas. Finalmente, para el contenido de ARMADILLO, no se censuró ningún mensaje en base a las preocupaciones anteriores.

Las actividades de ARMADILLO también proporcionaron información sobre aspectos de salud y bienestar relacionados con los adolescentes y los jóvenes donde se necesita más atención. Un hallazgo importante se relaciona con la presencia de la violencia en la vida de los adolescentes y jóvenes en este contexto. En las consultas comunitarias en la etapa 1, los participantes mencionaron espontáneamente la violencia de pareja como un tema importante y en el intercambio individual durante esas consultas comunitarias también se reveló violencia doméstica y abuso sexual en adolescentes, incluido a manos de parientes cercanos. En los grupos focales en la etapa 3, los mensajes de texto sobre violencia recibieron puntajes altos, lo que afirmó la demanda de información sobre el tema. Nuestros resultados confirmaron los hallazgos de estudios e informes anteriores en Perú, que encontraron altas tasas de violencia en el país (37–41). Un estudio en Perú encontró lo siguiente para casos de abuso sexual: en el 74% de los casos, los perpetradores eran parte del entorno cercano de la víctima; y en el 38% de los casos, los perpetradores eran miembros consanguíneos de la familia (42). Desafortunadamente, la violencia contra las mujeres, niños y adolescentes en el Perú se perpetúa generacionalmente y se reproduce por su aceptación cultural (43), mecanismos de denuncia débiles, falta de información sobre derechos, y desconfianza de la población hacia los proveedores e instituciones que deberían ofrecer protección frente a ella (38, 44). Teniendo en cuenta la desconfianza hacia los potenciales protectores y que a menudo son miembros de la familia los que practican o ejercen la violencia, los adolescentes afectados son extremadamente vulnerables (45). Estos hallazgos reafirman la importancia de incluir información sobre violencia en la plataforma ARMADILLO, para difundir información sobre lo que constituye abuso y violencia; para reforzar la información sobre los derechos y el valor de cada individuo como persona; y para brindar información sobre los servicios disponibles y generar confianza hacia estos servicios.

Adicionalmente, este estudio refuerza la investigación previa que muestra que los jóvenes peruanos carecen de conocimiento y acceso a información sobre la gama de métodos anticonceptivos, en especial, sobre los métodos más efectivos. Por ejemplo, la mayoría de los participantes informaron estar al tanto de los anticonceptivos orales de emergencia, pero no de las píldoras anticonceptivas regulares. Los participantes también describieron conceptos erróneos sobre los efectos secundarios significativos de varios métodos anticonceptivos. Nuestros hallazgos reafirman los resultados de estudios con jóvenes en otros PBMI, que sostienen varias creencias negativas sobre la anticoncepción, especialmente los ARLD (46-48). Las recomendaciones de la OMS para prevenir embarazos precoces requieren aumentar el uso de anticonceptivos entre las adolescentes que están sexualmente activas y no quieren quedar embarazadas (49). Una reciente revisión sistemática de las intervenciones para prevenir embarazos adolescentes en PBMI encontró que la provisión de métodos anticonceptivos fue altamente efectiva e incluso condujo a una disminución en las tasas de embarazo adolescente (50). Sin embargo, en una encuesta reciente con 2528 adolescentes en diferentes regiones del Perú sobre lo que aprenden durante la educación sexual brindada en la escuela, la anticoncepción fue uno de los temas menos discutidos y solo 5 de cada 10 participantes informaron haber aprendido dónde podían acceder a métodos anticonceptivos o cómo usarlos (51). Nuestros resultados, en el contexto actual de la política y las prácticas peruanas relacionadas con la SSR adolescente, demuestran la importancia de incluir información comprensiva y médicamente precisa sobre los métodos anticonceptivos y cómo acceder y usar estos métodos como parte del sistema ARMADILLO.

Nuestro trabajo tuvo algunas limitaciones. Con respecto a las consultas comunitarias, es posible que algunos temas de SSR de interés para adolescentes y jóvenes no hayan sido verbalizados debido a la presencia de adultos. Intentamos minimizar esta limitación al priorizar abiertamente la participación de los jóvenes sobre la de los adultos (que estuvieron presentes principalmente como observadores) y al proporcionar una opción confidencial para que los adolescentes y jóvenes también compartan temas de interés privadamente en lugar de públicamente. Con respecto a los grupos focales, pudimos haber obtenido resultados diferentes si hubiésemos dividido a los adolescentes y jóvenes por sexo. Sin embargo, quisimos promover el mayor diálogo posible, por lo tanto, incluimos hombres y mujeres en los mismos grupos. Ofrecimos oportunidades para contribuir individualmente para tratar de abordar las limitaciones al compartir que esto pudo haber ocasionado. Una limitación final fue que las actividades de la etapa 1 y la etapa 3 no fueron grabadas en audio, ya que el objetivo principal de esta fase formativa fue determinar los temas a incluir en el sistema de SMS, desarrollar y refinar una biblioteca de contenido de SMS y lograr este objetivo a través de participación individual y grupal, revisión en tiempo real, y refinamiento y consenso de las ideas compartidas en grupos. Sin embargo, las extensas notas del equipo y una sesión informativa entre el equipo (usando las notas y el material escrito de los participantes) después de cada sesión de actividad aseguraron una revisión detallada de la información compartida.

**Conclusiones**

Los resultados proporcionan información valiosa para el diseño participativo de las intervenciones de SSR para jóvenes y reafirman contundentemente el valor de incluir a adolescentes y jóvenes en todo el proceso de construcción de las intervenciones. Este estudio confirma la importancia de abordar los temas que son de mayor interés e importancia para los jóvenes. También proporciona información crítica sobre lo que los adolescentes y los jóvenes saben y cómo piensan, lo que nos permite comprender su perspectiva y literalmente hablar en su idioma, lo cual se integró en el sistema de SMS resultante. Finalmente, los resultados también indican futuros lineamientos para los esfuerzos programáticos, de investigación y de políticas con los jóvenes en Perú (y América Latina), en particular en torno a las normas de género, violencia interpersonal, acceso a información y servicios de SSR.

**Declaraciones**

Aprobación de ética y consentimiento de participación: no aplicable, ya que ya está incluido en el manuscrito.

Consentimiento para publicación - No aplicable

Disponibilidad de datos y material: no aplicable

**Conflicto de intereses**

Los autores declaran que no hay conflicto de intereses.

**Fondos**

El proyecto ARMADILLO fue financiado por el Programa Especial PNUD / UNFPA / UNICEF / OMS / Banco Mundial de Investigaciones, Desarrollo y Formación de Investigadores sobre Reproducción Humana a través de una donación a la Universidad Peruana Cayetano Heredia (UPCH) para llevar a cabo el Protocolo formativo ARMADILLO para el desarrollo de mensajes y las pruebas piloto correspondientes a la preparación de la plataforma ARMADILLO en ensayos de investigación en múltiples sitios. Mientras trabajó en este manuscrito, FG fue becaria académica de Kuskaya: un programa de capacitación interdisciplinaria para la innovación en salud global, financiado por el Centro Internacional Fogarty e implementado por UPCH y la Universidad de Washington.

El manuscrito representa solo las opiniones de los autores nombrados.

# Contribuciones de los autores

# MJH, LG y AMB desarrollaron el estudio y diseñaron las herramientas de recopilación de datos. MGC, MC, JPL y AMB analizaron e interpretaron la información recopilada durante las consultas comunitarias. FG, NL, MC y AMB analizaron e interpretaron la información recopilada durante los grupos focales. FG, NL y AMB escribieron el manuscrito. Todos los autores leyeron, hicieron contribuciones significativas y aprobaron el manuscrito final.

# Agradecimientos

# Muchas gracias a los adolescentes, jóvenes y adultos que trabajan con jóvenes que compartieron su tiempo, opiniones e ideas para ayudar a desarrollar el sistema de SMS ARMADILLO Perú.

# Lista de abreviaciones:

# ARMADILLO: Iniciativa de uso de mensajes de texto a celulares para mejorar resultados de amor y vida en adolescentes y jóvenes

# DHS: Encuesta Demográfica y de Salud Familia

# VIH: virus de inmunodeficiencia humana

# VIH / SIDA: virus de inmunodeficiencia humana / síndrome de inmunodeficiencia adquirida

# HRP: Programa Especial de Investigaciones, Desarrollo y Formación de Investigadores sobre Reproducción Humana del PNUD / UNFPA / UNICEF / OMS / Banco Mundial

# DIU: dispositivo intrauterino

# ARLD: anticonceptivos reversibles de larga duración

# PBMI: países de bajos y medianos ingresos

# MINEDU: Ministerio de Educación del Perú

# MINSA: Ministerio de Salud del Perú

# SMS: servicio de mensajería

# SSR: salud sexual y reproductiva

# ITS: infecciones de transmisión sexual

# UPCH: Universidad Peruana Cayetano Heredia

# OMS: Organización Mundial de la Salud

**Bibliografía**

1. Naciones Unidas, Departamento de Asuntos Económicos y Sociales, División de Población. World Population Prospects 2019 [Internet]. Datos personalizados adquiridos a través del sitio web; 2019. Disponible en: https://population.un.org/wpp/

2. INEI. Compendio Estadístico PERÚ 2015 [Internet]. [consultado el 28 de diciembre del 2017]. Disponible en: https://www.inei.gob.pe/media/MenuRecursivo/publicaciones_digitales/Est/Lib1253/compendio2015.html

3. INEI - Perú: Encuesta Demográfica y de Salud Familiar 2017 - Nacional y Regional [Internet]. [consultado el 12 de diciembre del 2018]. Disponible en: https://www.inei.gob.pe/media/MenuRecursivo/publicaciones_digitales/Est/Lib1525/index.html

4. Mendoza W, Subiría G. [Embarazo adolescente en Perú: su situación actual e implicaciones para las políticas públicas]. Rev Peru Med Exp Salud Publica. Julio de 2013; 30 (3): 471–9.

5. Fondo de Población de las Naciones Unidas (UNFPA). Embarazo adolescente en el Perú [Internet]. Lima, Perú: UNFPA; 2013 Jul. Disponible en: http://www.unfpa.org.pe/eaperu

6. Instituto Nacional de Estadística e Informática (INEI). Perú: Encuesta Demográfica y de Salud Familiar 2016 [Internet]. Lima, Perú: INEI; 2017. Disponible en: https://www.inei.gob.pe/media/MenuRecursivo/publicaciones_digitales/Est/Lib1433/index.html

7. Cárcamo CP, Campos PE, García PJ, Hughes JP, Garnett GP, Holmes KK, et al. Prevalencias de infecciones de transmisión sexual en adultos jóvenes y trabajadoras sexuales en Perú: una encuesta nacional basada en la población. Lancet Infect Dis. 2012 Oct; 12 (10): 765–73.

8. Minaya P. Situación del Embarazo Adolescente. Avances y Desafíos en la Salud Sexual y Reproductiva de las y los Adolescentes [Internet]. Diálogo por la Concertación: Prevención del Embarazo Adolescente; 2012 junio; Lima, Perú. Disponible en: http://www.unfpa.org.pe/historico/2012/indexJun2012B.htm

9. Fondo de Población de las Naciones Unidas D del P. Resultados de la supervisión defensiva a los servicios de salud diferenciados para la atención integral a adolescentes [Internet]. 2019. Disponible en: https://peru.unfpa.org/sites/default/files/pub-pdf/DP-UNFPA-servicios-diferenciados.pdf

10. Ministerio de Salud. Resolución Ministerial N ° 652-2016-MINSA [Internet]. [consultado el 20 de mayo de 2019]. Disponible en: https://www.gob.pe/institucion/minsa/normas-legales/191906-652-2016-minsa

11. Secretaría Nacional de la Juventud (SENAJU), Instituto Nacional de Estadística e Informática (INEI). Primera Encuesta Nacional de la Juventud Peruana (ENAJUV) 2011 [Internet]. Disponible en: http://www.unfpa.org.pe/publicaciones/publicacionesperu/SENAJU-INEI-ENAJUV-2011.pdf

12. Feyisetan B, Benevides R, Jacinto A, Mutombo, N. ¡Evaluación de los efectos de mCenas! SMS Educación sobre conocimiento, actitudes y autoeficacia relacionada con la anticoncepción en Mozambique [Internet]. Washington, DC: Proyecto de Evidencia para la Acción; 2015 Mar. Disponible en: https://www.e2aproject.org/wp-content/uploads/assessing-the-effects-of-mcenas.pdf

13. OneWorld Reino Unido. Lecciones aprendidas y resultados de la ampliación de Aprendizaje sobre la vida. Nigeria; 2009

14. Kaleidos Research, Centro Internacional de Salud Reproductiva. Acceso, Servicios, Conocimiento (ASK) - Youth Empowerment Alliance (YEA) [Internet]. Centro Internacional de Salud Reproductiva, Universidad de Gante; 2016. Disponible en: http://kaleidosresearch.nl/publication/ask-evaluation/

15. GSMA Mobile e inteligencia de desarrollo. YoungAfricaLive [Internet]. Disponible en: https://www.gsma.com/mobilefordevelopment/wp-content/uploads/2016/02/Case_Study_-YoungAfricaLive.pdf

16. Evidence to Action Project (E2A), USAID. Evaluar los efectos de mCenas! Educación de SMS sobre conocimiento, actitudes y autoeficacia relacionada con la anticoncepción entre jóvenes en Mozambique [Internet]. 2015 [consultado el 17 de julio del 2018]. Disponible en: https://www.popline.org/node/669119

17. Peters DH, Tran NT, Adam T. Investigación de implementación en salud: una guía práctica [Internet]. Alliance for Health Policy and System Research, Organización Mundial de la Salud; 2013. Disponible en: https://www.who.int/alliance-hpsr/alliancehpsr_irpguide.pdf

18. Al-Shorbaji N, Geissbuhler A. Estableciendo una base de evidencia para la salud electrónica: la prueba está en el budín. Bull Organo Mundial de la Salud. 2012 1 de mayo; 90 (5): 322-322A.

19. Kumar S, Nilsen WJ, Abernethy A, Atienza A, Patrick K, Pavel M, et al. Evaluación de tecnología móvil de salud: el taller de evidencia mHealth. Am J Prev Med. Agosto de 2013; 45 (2): 228–36.

20. Gonsalves L, L’Engle KL, Tamrat T, Plourde KF, Mangone ER, Agarwal S, et al. Estudio de Iniciativa de Acceso y Entrega Móvil para Adolescentes / Jóvenes para los Resultados de Amor y Vida (ARMADILLO): protocolo formativo para el desarrollo y pilotaje de la plataforma mHealth. Reprod Health. 2015 7 de agosto; 12: 67.

21. Instituto Nacional de Estadística e Informática (INEI). Serie Nacionales [Internet]. Lima, Perú: INEI; Disponible en: http://webapp.inei.gob.pe:8080/sirtod-series/

22. Instituto Nacional de Estadística e Informática (INEI). Perú: Encuesta Demográfica y de Salud Familiar 2015 [Internet]. Lima, Perú: INEI; 2016. Ava

Tabla 2: Nivel de interés de los participantes adolescentes y jóvenes en diferentes temas y subtemas de salud sexual y reproductiva (SSR) (Etapa 1), por grupo de edad, Lima, Yurimaguas y Ayacucho, Perú, 2015

|  | **Interés en los subtemas según la puntuación, rango de 3 (gran interés) a 0 (sin interés) ^1,2^** | | | |
| --- | --- | --- | --- | --- |
| **Temas (Puntaje promedio total)** | **Adolescentes de 13 a 17 años** | **Puntaje**  **promedio** | **Jóvenes de 18 a 24 años** | **Puntaje promedio** |
| **Identidad**  **(1,7)** | 🡹 Autoestima  🡹 Identidad sexual  ↓ Cambios físicos en varones  ↓ Cambios físicos en mujeres | (2,5)  (2,3)  (1,0)  (1,0) | 🡹 Exploración de la sexualidad  🡹 Menstruación  🡹 Orientación sexual  ↓ Cambios físicos en varones  ↓ Cambios físicos en mujeres | (3,0)  (2,7)  (2,3)  (1,0)  (1,0) |
| **Infecciones de Transmisión Sexual (ITS)**  **(1,7)** | 🡹 HIV  🡩 Prevención de ITS  ↓ Síntomas de ITS  O Herpes | (2,3)  (1,5)  (1,0)  (0,0) | 🡹 Síntomas de ITS  🡩 Prevención de ITS  🡩 Herpes  ↓ HIV | (2,0)  (2,0)  (2,0)  (1,0) |
| **Métodos barrera y anticonceptivos en SSR (para prevenir el embarazo, las ITS y el VIH) (1,6)** | 🡹 Abstinencia  🡩 Ritmo  ↓ Retiro | (2,3)  (1,5)  (0,5) | **🡹** Ritmo  🡹 Retiro  ↓ Abstinencia | (3,0)  (3,0)  (1,0) |
|  | 🡹 Anticonceptivo de emergencia (“píldora del día siguiente”)  🡹 Condón masculino  ↓ Dispositivos intrauterinos (DIUs)  ↓ Implantes | (3,0)  (3,0)  (0,8)  (0,8) | 🡹 Anticonceptivo de emergencia  🡹 Condón masculino  🡩 DIUs  O Implantes | (3,0)  (3,0)  (2,0)  (0,0) |
| **Relaciones interpersonales (1,3)** | 🡹 Comunicación familiar  (en general y relacionado a sexualidad)  🡹 Presión de grupo: relaciones sexuales  ↓ Exploración sexual con la pareja  O Atración y coqueteo | (3,0)  (2,5)  (0,8)  (0,0) | 🡹 “Amigos con derechos”  🡹 Presión de grupo: relaciones sexuales  O Atracción y coqueteo | (2,7)  (2,7)  (0,0) |
| **Prácticas sexuales**  **(1,3)** | 🡹 Sexo oral  🡹 Sexo transaccional  🡩 Masturbación  O Sexo sin penetración  O Masturbación mutua | (2,5)  (2,3)  (2,0)  (0,0)  (0,0) | 🡹 Sexo transaccional  🡹 Sexo anal  🡹 Sexo oral  🡩 Masturbación  ↓ Sexo sin penetración  ↓ Masturbación mutua | (2,7)  (2,3)  (2,3)  (2,0)  (0,3)  (0,3) |
| **Violencia**  **(1,2)** | 🡹 Violencia en la pareja  🡹 Abuso sexual y violación  O Cómo prevenir la violencia | (2,5)  (2,3)  (0,0) | 🡩 Violencia en la pareja  ↓ Abuso sexual y violación  ↓ Cómo prevenir la violencia | (2,0)  (0,4)  (0,3) |
| **Servicios de salud**  **(1,1)** | 🡩 Servicios de SSR  ↓ Acceso a servicios de salud  O Seguro de salud | (1,3)  (0,3)  (0,0) | 🡹 Acceso a servicios de salud  🡩 Salud mental / servicios psicológicos  O Seguro de salud | (3,0)  (2,0)  (0,0) |
| **Embarazo**  **(0,7)** | 🡹 Embarazo  O Signos de embarazo  O Complicaciones de embarazo | (3,0)  (0,0)  (0,0) | 🡹 Embarazo  ↓ Signos de embarazo  O Complicaciones de embarazo | (3,0)  (1,0)  (0,0) |
| **Derechos y políticas de SSR**  **(0,6)** | 🡹 Aborto  O Derecho a la información sobre SSR  O Derecho a la atención en salud | (2,3)  (0,0)  (0,0) | 🡹 Aborto  ↓ Derecho a la información sobre SSR  ↓ Derecho a la atención en salud | (3,0)  (0,3)  (0,3) |
| **Alcohol y drogas**  **(0,2)** | 🡩 Tipos de drogas  ↓ Presión de grupo: uso de alcohol y drogas | (1,3) (0,8) | ↓ Presión de grupo: uso de alcohol y drogas  O Tipo de drogas | (0,4)  (0,0) |

1 Los puntajes de la tabla se basan en las calificaciones de los adolescentes y los jóvenes de los mensajes de texto SMS durante las consultas comunitarias de la Etapa 1. Los participantes a) mencionaron espontáneamente los temas y subtemas de interés y b) calificaron su interés en los subtemas incluidos en los proyectos de SSR en otros entornos. La escala de calificación varía de 0 (sin interés) a 3 (de gran interés). Para los subtemas, el puntaje representa la calificación promedio del subtema en las seis reuniones. Para los temas, el puntaje representa el promedio de los puntajes para los subtemas incluidos en el tema. Solo los subtemas con puntajes más altos y más bajos se incluyen en la Tabla. / 2 Los símbolos a la izquierda de los subtemas significan lo siguiente: O - Sin interés (igual a cero);↓ - De poco interés (puntuación entre 0,1 y 1,0);🡩 - De interés (Puntuación entre 1,1 y 2,0); y 🡹- De gran interés (puntaje entre 2,1 y 3,0),

Tabla 4: Ejemplos de versiones preliminares y finales de los mensajes de texto SMS de diferentes dominios y subdominios de la plataforma ARMADILLO Perú SMS

| **Dominios y Subdominios** | **Ejemplos de mensajes de texto^1^ del subdominio, versión borrador creada por el equipo durante la etapa 2** | | **Ejemplos de mensajes de texto^1^ del subdominio, versión final después de los comentarios de los participantes durante la etapa 3** |
| --- | --- | --- | --- |
| **¿Quién soy?**  *Pubertad* | "En la adolescencia el cuerpo cambia mucho, se llama pubertad. Es normal que en algunas personas los cambios sean más marcados que en otras". | | "En la adolescencia el cuerpo tiene muchos cambios físicos y emocionales, se llama pubertad. ¡Todos NO pasamos por los mismos cambios!" |
| **¿Quién soy?**  *Identidad sexual* | "Todos nacemos con un sexo (hombre o mujer), pero no todos nos sentimos bien con ello. Es normal. Sólo nosotros mismos sabemos lo que sentimos”. | | "Todos nacemos con un sexo (hombre o mujer) pero no todos nos sentimos bien con ello. ¡Es normal! La decisión es solo tuya". |
| **¿Quiénes me cuidan?**  *Familia* | “A nadie le enseñan a ser padre. A veces quieren ayudarte y darte su amor, pero no saben cómo hacerlo. Busca el momento adecuado". | | “A nadie le enseñan a ser padre. A veces quieren ayudarte y darte su amor, pero no siempre saben cómo hacerlo. ¡Pídeles apoyo y te apoyarán!” |
| **¿Quiénes me cuidan?**  *Amigos* | "Un amigo no te presiona a hacer algo que no quieres hacer. Un amigo respeta tu decisión". | | "Un amigo NO te presiona a hacer algo que no quieres hacer. Un amigo respeta tu decisión". |
| **¿Quiénes me cuidan?**  *Parejas* | "Una relación saludable es aquella que te hace feliz. Una relación que te hace sentir triste no vale la pena". | | "Una relación saludable es la que te hace feliz, donde hay un aprecio mutuo, fidelidad, respeto y buena comunicación". |
| **¿Cómo disfruto?**  *Con alguien más* | "Antes de tener sexo con alguien, la confianza y la aceptación son clave!" | | "Antes de tener sexo con alguien, debes sentirte cómodo con esa persona y también contigo mismo. ¡No olvides de cuidarte!" |
| **¿De qué me cuido?**  *El embarazo* | "Un embarazo puede darse en cualquier momento. Incluso en tu primera relación sexual. ¿Sí? Sí, es cierto. ¡Cuídate!" | | "Un embarazo puede ocurrir en cualquier momento. Incluso durante tu primera relación sexual. Es cierto. ¡Ten cuidado!" |
| **¿De qué me cuido?**  ITS | "Si has tenido sexo sin condón, hazte una prueba de ITS - infección de transmisión sexual. Si tu pareja tiene o tuvo una ITS, con más razón". | | "Si has tenido relaciones sexuales sin condón, hazte una prueba de ITS (infección de transmisión sexual). Infórmate sobre el tema. La prueba es GRATUITA". |
| **¿Cómo me cuido?**  *Abstinencia* | "Decidir no tener relaciones sexuales hasta estar preparado y encontrar a la persona correcta es una decisión responsable". | | "Decidir no tener relaciones sexuales hasta encontrar a la persona correcta y estar preparado es una decisión responsable. ¡No hay prisa!" |
| **¿Cómo me cuido**  *Condón* | "El condón no solo es para sexo vaginal, también es para sexo anal. Recuerda, un nuevo condón con cada penetración y solo UN condón a la vez". | | "El condón no sólo se usa en el sexo vaginal, también en el sexo anal y oral. ¡Recuerda usar un condón nuevo para cada erección! ¡USA UNO POR VEZ!" |
| **¿Cómo me cuido?**  *Implantes y DIU* | "Los implantes y dispositivos intrauterinos-DIUs son los métodos MAS efectivos para prevenir embarazos. ¡Y son apropiados para adolescentes!". | "El implante y el dispositivo intrauterino o DIU son los métodos MÁS EFECTIVOS para prevenir el embarazo. ¡Y son adecuados para adolescentes!" | |
| **Lo que todos callamos**  *Aborto* | "Hacerse un aborto en un lugar que ofrece solucionar el "Atraso menstrual", puede terminar con problemas de salud o la muerte de la gestante". | "Realizarse un aborto en un lugar clandestino puede causar problemas de salud o incluso la muerte". | |
| **Lo que todos callamos**  *Violencia* | "Más te pego, más te quiero? NO. Nadie que te pega te puede querer. Nadie que te haga sufrir, te puede amar". | "Cuanto más te golpeo, más te amo? No es correcto. Nadie que te pegue te puede querer. Nadie que te haga sufrir puede amarte. ¡VALÓRATE!" | |

# ^1^ ^Cada subdominio tenía de 2 a 10 mensajes de texto asociados.^
